# Adjusting mobile phone data to account for children’s travel and the impact on measles dynamics in Zambia

**DOI:** 10.1093/aje/kwae304

**Published:** 2024-08-27

**Authors:** Natalya Kostandova, Christine Prosperi, Simon Mutembo, Chola Nakazwe, Harriet Namukoko, Bertha Nachinga, Gershom Chongwe, Innocent Chilumba, Elliot N Kabalo, Kabondo Makungo, Kalumbu H Matakala, Gloria Musukwa, Mutinta Hamahuwa, Webster Mufwambi, Japhet Matoba, Irene Mutale, Edgar Simulundu, Phillimon Ndubani, Alvira Z Hasan, Shaun A Truelove, Amy K Winter, Andrea C Carcelen, Bryan Lau, William J Moss, Amy Wesolowski

**Affiliations:** Department of Epidemiology, Johns Hopkins University Bloomberg School of Public Health, Baltimore, MD 21205, United States; Department of International Health, Johns Hopkins University Bloomberg School of Public Health, Baltimore, MD 21205, United States; Department of International Health, Johns Hopkins University Bloomberg School of Public Health, Baltimore, MD 21205, United States; Information, Research and Dissemination, Zambia Statistics Agency, Lusaka 1010, Zambia; Population and Social Statistics, Zambia Statistics Agency, Lusaka 1010, Zambia; Information, Research and Dissemination, Zambia Statistics Agency, Lusaka 1010, Zambia; Tropical Diseases Research Centre, Ndola 10101, Zambia; Biomedical Sciences Department, Tropical Diseases Research Centre, Ndola 10101, Zambia; Zambia Information and Communications Technology Authority, Lusaka 36871, Zambia; Zamtel, Lusaka 37000, Zambia; Clinical Research Department, Macha Research Trust, Choma 10101, Zambia; Administration, Macha Research Trust, Choma 10101, Zambia; Clinical Research Department, Macha Research Trust, Choma 10101, Zambia; Administration, Tropical Diseases Research Centre, Ndola 10101, Zambia; Molecular Biology Department, Macha Research Trust, Choma 10101, Zambia; Biomedical Sciences Department, Tropical Diseases Research Centre, Ndola 10101, Zambia; Administration, Macha Research Trust, Choma 10101, Zambia; Administration, Macha Research Trust, Choma 10101, Zambia; Department of International Health, Johns Hopkins University Bloomberg School of Public Health, Baltimore, MD 21205, United States; Department of Epidemiology, Johns Hopkins University Bloomberg School of Public Health, Baltimore, MD 21205, United States; Department of International Health, Johns Hopkins University Bloomberg School of Public Health, Baltimore, MD 21205, United States; Department of International Health, Johns Hopkins University Bloomberg School of Public Health, Baltimore, MD 21205, United States; Department of Epidemiology and Biostatistics, University of Georgia, Athens, GA 30606, United States; Department of International Health, Johns Hopkins University Bloomberg School of Public Health, Baltimore, MD 21205, United States; Department of Epidemiology, Johns Hopkins University Bloomberg School of Public Health, Baltimore, MD 21205, United States; Department of Epidemiology, Johns Hopkins University Bloomberg School of Public Health, Baltimore, MD 21205, United States; Department of International Health, Johns Hopkins University Bloomberg School of Public Health, Baltimore, MD 21205, United States; Department of Epidemiology, Johns Hopkins University Bloomberg School of Public Health, Baltimore, MD 21205, United States

**Keywords:** mobility, bias, mobile phone data, call data records, measles, children, infectious disease modeling

## Abstract

Models of measles transmission can be used to identify areas of high risk to tailor immunization strategies. Estimates of spatial connectivity can be derived from data such as mobile phone records, but it is not clear how this maps to the movement of children who are more likely to be infected. Using travel surveys across 2 districts in Zambia and national mobile phone data, we compared estimates of out-of-district travel for the population captured in the mobile phone data and child-specific travel from travel surveys. We then evaluated the impact of unadjusted and adjusted connectivity measures on simulated measles virus introduction events across Zambia. The number of trips made by children from the travel survey was 3 to 5 times lower than the general population estimates from mobile phone data. This decreased the percentage of districts with measles virus introduction events from 78% when using unadjusted data to 51% to 64% following adjustment. Failure to account for age-specific heterogeneities in travel estimated from mobile phone data resulted in overestimating subnational areas at high risk of introduction events, which could divert mitigation efforts to districts that are at lower risk.

## Introduction

Despite progress toward achieving measles control and elimination, recent global outbreaks of measles and disruptions to vaccination services as a result of the pandemic suggest that progress toward elimination has stalled.^1^ In 2019, there were more than 870 000 reported measles cases and 207 500 estimated deaths, the highest burden of disease in almost 3 decades.^2^ Although an immunizing and effective vaccine is available, high vaccination coverage is needed to maintain control and elimination,^3^^,^^4^ with even small decreases in coverage resulting in an increased risk of outbreaks. Achieving and maintaining high coverage remains a challenge, exacerbated by the COVID-19 pandemic, which resulted in significant disruptions to vaccination programs.^5^

In many settings, a combination of both routine childhood vaccination and additional campaigns, primarily preventative supplementary immunization activities (SIAs) and reactive SIAs during an outbreak, is needed to achieve high measles vaccine coverage.^6^ While SIAs have historically been conducted nationally for specified age groups, their high costs and strain on resources have shifted focus toward targeting areas at a high risk of an outbreak.^4^^,^^7^ Outbreaks occur in locations where there are (1) sufficiently large susceptible populations, measured indirectly using administrative vaccination and case data and more recently directly using serological data, and (2) importations of infected measles cases, inferred from travel data. Mobile phone call records or census migration data have been primarily used to quantify travel relevant to measles virus introduction events.^8^^-^^12^ However, mobility estimates from these data sources may capture travel of a particular subpopulation that is not necessarily the most important for measles virus transmission. For example, mobile phone data are restricted to data from individuals who own or carry a phone, and there is differential ownership by age, sex, socioeconomic, and psychosocial status.^13^^-^^22^ Thus, if population travel patterns are heterogeneous by mobile phone ownership, these data may lead to biases in mobility estimates, resulting in biased estimates of outbreak risk. Since children are at the highest risk of measles virus infection, age biases in mobility estimates may have a particularly strong impact on estimates of population-level mobility of those most likely to be infectious. While some studies suggest that estimates of adult travel patterns are robust to demographic and socioeconomic biases in phone ownership,^23^ it remains unclear to what extent mobile phone data capture the movement of children or what methods can be used to adjust these patterns to better account for this subpopulation.

Zambia has made substantial progress on the path to measles elimination.^24^ Few measles cases have been reported between 2014 and 2019^25^ as a result of historically high routine coverage of the first dose of measles-containing vaccine (MCV1) and nationwide SIA campaigns taking place every 4 years since 2003. However, coverage of the second dose of measles-containing vaccine (MCV2) has consistently lagged behind,^26^ and since 2020, measles vaccination coverage has declined (MCV1 coverage 96% in 2020 and 90% in 2021^26^). Recent and ongoing local outbreaks since 2021 suggest that progress toward achieving measles control is stagnating or regressing.^27^ For Zambia to achieve measles elimination, it will be critical to identify and target priority areas for improved SIA and routine immunization activities.

In this analysis, we aimed to assess subnational risk of measles outbreaks by incorporating less biased estimates of population travel patterns. This included estimating multiple connectivity measures: 1 from mobile phone data alone that does not adjust for biases in phone ownership and 3 from mobile phone data and 2-district travel surveys that adjust for biases in phone ownership and children’s travel patterns. We then constructed a spatially explicit model of measles transmission throughout the country and assessed how the risk of introduction, the median time to introduction and outbreak, and the impact of reactive SIAs changed when travel patterns were estimated using the unadjusted and adjusted connectivity measures.

## Methods

We quantified connectivity between districts in Zambia using 2 data sources: mobile phone call records and a travel survey from 2 districts in Zambia.

### Mobile phone call records

**Figure 1:**
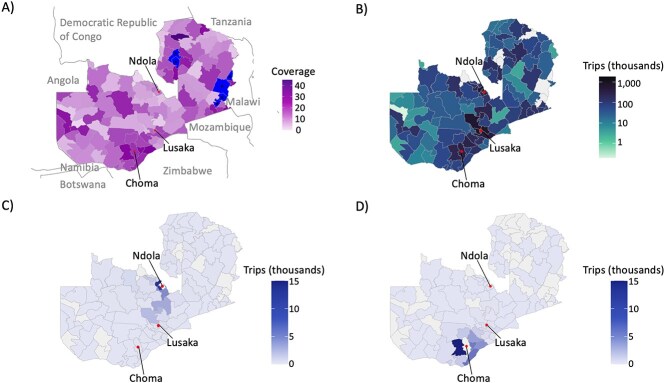
District-level mobile phone data from the Zambia Telecommunications Company Limited (Zamtel) network provider, collected between March 1, 2020, and December 30, 2020. (A) District-level estimated Zamtel coverage (estimated coverage in Choma was 19% and 11% in Ndola). Coverage is calculated as the maximum number of subscribers in a month over a 9-month period (provided by Zamtel) and divided by the estimated population size in 2020. Districts with an asterisk (*) are those with no reported coverage. (B) The average number of monthly trips taken into each district based on Zamtel mobile phone data. (C) The average number of monthly trips taken from Ndola District, using Zamtel mobile phone data. Ninety-nine districts were destinations for trips out of Ndola District. (D) The average number of monthly trips taken from Choma District, using Zamtel mobile phone data. Eighty-one districts were destinations for trips out of Choma District.

Mobile phone data were obtained from Zambia Telecommunications Company Limited (Zamtel), 1 of 3 major mobile phone operators in Zambia. Data were provided from March 1, 2020, to December 30, 2020. Movement was estimated based on primary mobile phone tower location used per SIM card per day with counts of trips between districts describing the number of SIM cards for which primary mobile phone towers were in different districts on subsequent days. For all records where the primary mobile phone tower remained in the same district, no movement was assumed. We used the total number of trips within and between districts to calculate the probability of remaining in each district and the probability of travel between each set of districts. In total, 109 of 115 districts in Zambia had subscribers within these data sets (see Figure 1A).

### Travel survey

A travel survey was embedded in a community measles serological survey in Choma and Ndola Districts (see Figure S1). Data collection occurred between March and June 2022 and was carried out following a multistage stratified cluster methodology (see Appendix S1). We enrolled 765 caretakers of younger children (1-4 years old), 1466 caretakers of older children (5-14 years old), and 1346 adults (individuals 15 years and older) (see Table S1). The travel survey included sociodemographic information on households and respondents, respondents’ mobile phone ownership, and information on overnight travel in the 2 months before the survey, including district(s) traveled to, the reason for travel, and whether the respondent was accompanied by anyone, including children. To compare to the mobile phone data, monthly estimates of trip counts were generated. Travel survey estimates presented in Table 1 are weighted estimates to account for sampling and nonresponse. All *P* values reported are 2-sided.

**Table 1:** Results of travel survey in Choma and Ndola districts, Zambia, 2022.

		**Choma**				**Ndola**		
	**Overall**	**Caretakers of children 1 to 4 years**	**Caretakers of children 5 to 14 years**	**Adults**	**Overall**	**Caretakers of children 1 to 4 years**	**Caretakers of children 5 to 14 years**	**Adults**
**Owns a mobile phone**								
Proportion	69.4%	59.9%	70.4%	65.1%	79.2%	71.1%	76.1%	76.5%
*Sample size*	*2055*	*444*	*834*	*777*	*1521*	*321*	*631*	*569*
**Had an overnight trip in the past 2 months**								
*Overall*								
Proportion with an overnight trip	19.5% [14.0%; 25.0%]	21.8% [14.0%; 29.6%]	19.5% [13.7%; 25.3%]	18.4% [13.2%; 23.5%]	10.9% [7.6%; 14.2%]	7.6% [3.5%; 11.6%]	9.6% [5.9%; 13.4%]	13.6% [6.8%; 20.3%]
*Sample size*	*2055*	*444*	*834*	*777*	*1521*	*321*	*631*	*569*
*Among mobile phone owners*								
Proportion with an overnight trip	19.7% [11.8%; 26.6%]	22.3% [12.5%; 32.2%]	18.3% [12.1%; 24.5%]	19.8% [14.1%; 25.4%]	9.1% [3.9%; 14.2%]	9.5% [4.6%; 14.4%]	8.7% [5.8%; 14.1%]	13.2% [7.5%; 20.0%]
*Sample size*	*1427*	*290*	*603*	*534*	*1204*	*242*	*515*	*447*
*Among those who do not own a mobile phone*								
Proportion with an overnight trip	19.2% [13.7%; 25.7%]	20.9% [12.2%; 29.6%]	22.3% [12.4%; 32.3%]	15.8% [7.9%; 23.7%]	11.5% [8.2%; 14.8%]	1.6% [0%; 4.0%]	9.9% [2.2%; 15.2%]	13.7% [2.1%; 24.4%]
*Sample size*	*628*	*154*	*231*	*243*	*317*	*79*	*116*	*122*
**Proportion overnight trips within district (among those who had overnight travel)**								
*Overall*								
Proportion overnight trips within district	66.1% [55.7%; 76.5%]	67.4% [56.9%; 78.0%]	67.1% [56.3%; 77.8%]	64.3% [50.5%; 78.1%]	59.9% [39.7%; 80.1%]	40.9% [18.4%; 63.4%]	43.8% [28.5%; 59.1%]	73.5% [50.5%; 96.5%]
*Sample size*	*519*	*116*	*225*	*178*	*191*	*35*	*78*	*78*
*Among mobile phone owners*								
Proportion overnight trips within district	56.4% [46.5%; 66.3%]	56.7% [40.6%; 72.8%]	59.7% [49.7%; 69.7%]	53.3% [40.6%; 65.9%]	53.0% [34.6%; 71.4%]	42.7% [19.7%; 65.8%]	41.6% [22.1%; 61.0%]	63.7% [39.1%; 88.3%]
*Sample size*	*374*	*80*	*163*	*131*	*165*	*33*	*66*	*66*
*Among those who do not own a mobile phone*								
Proportion overnight trips within district	83.7% [74.3%; 93.1%]	82.6% [73.4%; 91.7%]	81.1% [66.8%; 95.4%]	87.2% [74.5%; 100.0%]	82.9% [62.5%; 103.3%]	0%	52.9% [21.4%; 84.5%]	96.1% [87.2%; 105.1%]
*Sample size*	*145*	*36*	*62*	*47*	*26*	*2*	*12*	*12*
**Travel with others (among those who had overnight travel)**								
*Overall*								
Proportion traveled with others	54.9% [41.8%; 68.0%]	68.5% [55.8%; 81.2%]	52.9% [40.0%; 65.8%]	48.0% [30.5%; 65.5%]	49.7% [38.8%; 60.7%]	63.3% [43.1%; 83.6%]	64.8% [51.6%; 78.1%]	38.1% [28.1%; 48.2%]
*Sample size*	*519*	*116*	*225*	*178*	*191*	*35*	*78*	*78*
*Among mobile phone owners*								
Proportion traveled with others	45.1% [36.9%; 53.3%]	64.5% [52.2%; 76.7%]	47.3% [37.6%; 57.0%]	32.3% [20.5%; 44.1%]	52.3% [41.7%; 64.2%]	61.7% [40.3%; 83.0%]	63.6% [50.7%; 76.4%]	43.3% [24.8%; 61.8%]
*Sample size*	*374*	*80*	*163*	*131*	*165*	*33*	*66*	*66*

**Table 1:** Continued

		**Choma**				**Ndola**		
	**Overall**	**Caretakers of children 1 to 4 years**	**Caretakers of children 5 to 14 years**	**Adults**	**Overall**	**Caretakers of children 1 to 4 years**	**Caretakers of children 5 to 14 years**	**Adults**
*Among those who do not own a mobile phone*								
Proportion traveled with others	72.8% [53.1%; 92.4%]	74.1% [52.2%; 96.1%]	63.5% [37.6%; 89.5%]	80.6% [63.1%; 98.2%]	39.1% [0%; 78.8%]	100%^a^	70.1% [39.7%; 100%]	26.3% [0%; 66.3%]
*Sample size*	*145*	*36*	*62*	*47*	*26*	*2*	*12*	*12*
**Travel with children (among those that had overnight travel)**								
*Overall*								
Proportion travel with children	26.4% [18.0; 34.8%]	42.5% [31.4%; 53.6%]	23.9% [15.6%; 32.2%]	18.4% [7.7%; 29.0%]	18.8% [7.9%; 29.8%]	41.2% [23.2%; 59.1%]	31.2% [19.4%; 42.9%]	6.3% [0%; 13.4%]
*Sample size*	*519*	*116*	*225*	*178*	*191*	*35*	*78*	*78*
*Among mobile phone owners*								
Proportion travel with children	21.9% [16.1%; 27.6%]	43.7% [33.3%; 54.2%]	18.9% [12.0%; 25.8%]	12.3% [4.4%; 20.2%]	20.3% [11.1%; 29.6%]	41.4% [24.5%; 58.4%]	33.4% [21.2%; 45.6%]	4.6% [0%; 9.4%]
*Sample size*	*374*	*80*	*163*	*131*	*165*	*33*	*66*	*66*
*Among those who do not own a mobile phone*								
Proportion travel with children	34.7% [19.6%; 49.8%]	40.8% [16.5%; 65.1%]	33.5% [15.6%; 51.3%]	31.0% [15.1%; 46.9%]	21.9% [0%; 30.4%]	35.0% [0%; 99.1%]	22.0% [5.4%; 38.5%]	10.4% [0%; 29.4%]
*Sample size*	*145*	*36*	*62*	*47*	*26*	*2*	*12*	*12*
**Number of children traveled with (among those with overnight trip)**								
*Overall*								
Number of children traveled with	0.31 [0.23; 0.40]	0.48 [0.35; 0.62]	0.29 [0.21; 0.38]	0.22 [0.10; 0.34]	0.34 [0.12; 0.55]	0.59 [0.33; 0.86]	0.48 [0.30; 0.66]	0.19 [–0.09; 0.47]
*Sample size*	*519*	*116*	*225*	*178*	*191*	*35*	*78*	*78*
*Among mobile phone owners*								
Number of children traveled with	0.27 [0.19; 0.34]	0.51 [0.39; 0.64]	0.24 [0.16; 0.32]	0.09 [0.04; 0.27]	0.31 [0.17; 0.46]	0.60 [0.33; 0.87]	0.50 [0.32; 0.69]	0.43 [0; 0.19]
*Sample size*	*374*	*80*	*163*	*131*	*165*	*33*	*66*	*66*
*Among those who do not own a mobile phone*								
Number of children traveled with	0.39 [0.24; 0.55]	0.44 [0.17; 0.72]	0.40 [0.22; 0.58]	0.35 [0.18; 0.53]	0.42 [–0.24; 1.08]	0.35 [–0.29; 0.99]	0.39 [0.04; 0.74]	0.09 [–0.48; 1.34]
*Sample size*	*145*	*36*	*62*	*47*	*26*	*2*	*12*	*12*
**Number of children traveled with (among those that traveled with others)**								
*Overall*								
Number of children traveled with	0.57 [0.45; 0.69]	0.71 [0.51; 0.90]	0.56 [0.38; 0.73]	0.46 [0.29; 0.63]	0.68 [0.33; 1.03]	0.94 [0.48; 1.39]	0.74 [0.52; 0.96]	0.51 [–0.20; 1.22]
*Sample size*	*270*	*72*	*120*	*78*	*101*	*22*	*51*	*28*
*Among mobile phone owners*								
Number of children traveled with	0.59 [–0.28; 2.42]	0.80 [0.59; 1.01]	0.50 [0.33; 0.68]	0.44 [0.20; 0.77]	0.59 [0.30; 0.88]	0.98 [0.48; 1.48]	0.79 [0.57; 1.01]	0.21 [–0.05; 0.47]
*Sample size*	*174*	*49*	*80*	*45*	*87*	*20*	*43*	*24*

**Table 1:** Continued

		**Choma**				**Ndola**		
	**Overall**	**Caretakers of children 1 to 4 years**	**Caretakers of children 5 to 14 years**	**Adults**	**Overall**	**Caretakers of children 1 to 4 years**	**Caretakers of children 5 to 14 years**	**Adults**
*Among those who do not own a mobile phone*								
Does not own a mobile phone	0.54 [0.40; 0.69]	0.59 [0.29; 0.90]	0.63 [0.36; 0.90]	0.48 [0.24; 0.64]	1.07 [–0.28; 2.42]	0.35 [–0.29; 0.99]	0.55 [0.04; 1.06]	1.63 [–1.06; 4.33]
*Sample size*	*96*	*23*	*40*	*33*	*14*	*2*	*8*	*4*
**Distance traveled (among those with an overnight trip outside district)**								
*Overall*								
Distance traveled	93.4 km [82.6; 104.3]	85.3 km [70.5; 100.1]	90.4 km [78.2; 102.5]	100.8 km [79.9; 121.7]	271.9 km [226.9; 316.9]	249.4 km [158.5; 340.3]	268.5 km [196.7; 340.4]	288.3 km [216.5; 360.1]
*Sample size*	*199*	*43*	*84*	*72*	*103*	*20*	*46*	*37*
*Among mobile phone owners*								
Distance traveled	98.6 km [86.7; 110.5]	92.9 km [74.7; 111.1]	96.3 km [82.1; 110.4]	103.3 km [80.2; 126.3]	269.9 km [223.3; 316.6]	234.6 km [135.3; 334.0]	277.9 km [217.5; 338.3]	282.0 km [208.2; 355.8]
*Sample size*	*166*	*32*	*70*	*64*	*95*	*18*	*41*	*36*
*Among those who do not own a mobile phone*								
Distance traveled	68.8 km [50.2; 87.5]	58.5 km [44.4; 72.5]	67.6 km [50.2; 85.0]	82.1 km [38.4; 125.9]	288.7 km [98.1; 479.2]	420.3 km [419.5; 421.0]	223.9 km [6.8; 441.1]	421.0 km [421.0; 421.0]
*Sample size*	*33*	*11*	*14*	*8*	*8*	*2*	*5*	*1*

All results except the number of participants (sample size) are weighted. Brackets indicate the 95% confidence interval.

^a^Sample size too low to obtain confidence interval.

### Estimating connectivity between districts using mobile phone and travel survey data

We considered both unadjusted (raw) and adjusted (integrating ownership and differential travel from the survey) estimates of mobility from the mobile phone data. For the unadjusted data, the average number of monthly trips between districts was normalized by the origin district. For the adjusted data, we rescaled the unadjusted data to estimate the expected number of trips taken by children younger than 15 years using the travel survey data. For the 2 districts with survey data, the total population was distributed among the number falling within each respondent group (based on age and caretaker status) and then by mobile phone ownership, trips taken, and number of accompanying children (see Appendix S2). This provided an estimate of the number of trips taken by children over the course of 2 months adjusted for mobile phone ownership and travel by group. Values were divided by 2 and by an estimated number of children in each district to estimate the monthly probability of children’s travel. Since the survey was only conducted in 2 districts, we scaled by the Choma, Ndola, or a “mixed” value based on the similarities of population density to each location*.* Values were capped at 0.9956, the maximum probability of remaining within a district in the unadjusted mobility matrix, and normalized by the origin district.

As mobile phone coverage was not nationwide, we also fit a range of mobility models to the connectivity measures (see Appendix S3) using the R package mobility.^28^ The best-fitting model was a departure-diffusion (exponential gravity) model, which models the probability of travel from district $i$as a function of 2 probabilities—the probability of leaving the district (probability of departure) and the probability of traveling to district $j$ for those who left the district (diffusion process). A comprehensive description of the departure-diffusion and other explored models is available elsewhere.^29^^,^^30^ We estimated parameters for all districts with mobile phone data (*n =* 107), predicted the mean number of trips for all districts (*n =* 115) using the fit model (see Table S2), and the adjusted version (as above).

### Simulating measles virus transmission among districts in Zambia

We used a spatial discrete-time stochastic model to simulate measles virus transmission dynamics. The model includes 5 epidemiologic classes: Maternal Immunity, Susceptible, Infected, Vaccinated, and Recovered. We allowed for differential effectiveness of vaccination depending on the dose and mode of administration (e.g., via routine vs campaign activities). Namely, an individual receiving a first dose of measles-containing vaccine either became immune (moved to the Recovered compartment) or remained unprotected (moving to the *V1C* compartment for those receiving their dose via campaign or the *V1R* compartment for those vaccinated via routine administration). Individuals receiving a second dose (regardless of mode of administration) became immune. A full description of the model is provided in Appendix S4. 

To simulate conditions similar to Zambia, we began simulations one generation time before the 2020 SIA (week 34 of 2020), with districts with initial infections being those with recently reported outbreaks and reflective of the magnitude of the outbreak by location, as obtained from a fever rash line list of cases^31^ (initially infected districts: Chirundu = 10, Lunte = 5, and Mafinga = 5). Each simulation ran for 1 year to capture the early dynamics of a measles outbreak in a highly vaccinated population. We ran 100 simulations per measure of connectivity and compared results based on the proportion of districts with at least 1 introduction event (upper limit of incident cases > 1 across simulations) and the median time to this event. We also assessed district characteristics for locations with an introduction occurring in at least 10% of simulations. We fit 2 separate logistic regression models. In the first model, the dependent variable was defined as locations with a high probability (≥ 10%) of having an introduction event only when the modeled number of trips was used (compared to when the raw number of trips was used). In the second model, the dependent variable was locations with a high probability of having an introduction event only when the unadjusted predicted number of trips was used (compared to the adjusted number of trips using a “mixed” approach). In both models, covariates included district characteristics (2020 population size, 2018 MCV1 coverage, and number of trips into another district from mobile phone data). Adjusted odds ratios were calculated along with 95% confidence intervals. Association was considered statistically significant if the 2-tailed *P* value was < .05.

### Evaluating the effectiveness of a reactive measles vaccination campaign

We modeled a reactive measles vaccination campaign. Consistent with the World Health Organization’s outbreak response strategy,^6^ we assumed that a vaccination campaign would occur 4 weeks after a district reached 3 cumulative measles cases. Coverage was set to 80% of the susceptible population, a value higher than the 2020 SIA but in line with previous SIA coverage levels. As an outcome of interest, for each of the unadjusted and adjusted approaches, we considered the relative reduction in cumulative infections following vaccination. As a sensitivity analysis, we also considered a vaccination campaign in the 3 provinces with initial infections (Lusaka, Northern, and Eastern) that took place 8 weeks after the start of the simulation. This could be a viable option for a campaign triggered by the first reported cases if there were concerns about the underreporting of cases in other districts within the provinces with ongoing transmission and mobility between districts. Coverage was set to 95%, similar to SIA coverage before the COVID-19 pandemic.

A graphical summary of the methods is provided in Appendix S5.

### Ethics approval

The study was approved by the Johns Hopkins Bloomberg School of Public Health Institutional Review Board (IRB) (IRB00011162; IRB00018265) and the Zambia National Health Research Authority (IRB00002911).

## Results

**Figure 2:**
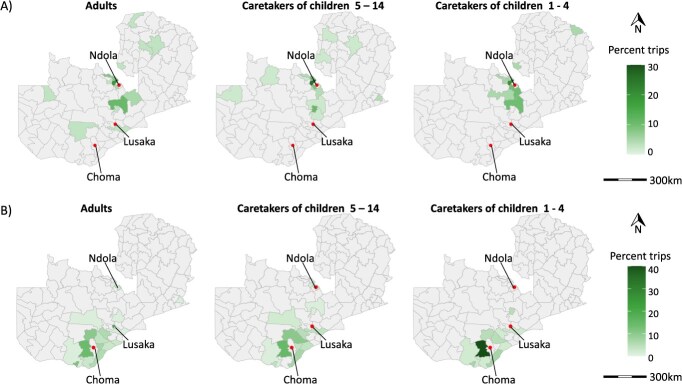
Distribution of overnight trips out of the district of origin based on the travel survey conducted in Choma and Ndola Districts for the 2 months preceding the travel survey. (A) Distribution of out-of-district trips taken from Ndola District. A total of 20 districts were named by participants as destinations for overnight trips. (B) Distribution of out-of-district trips from Choma District. Respondents named 26 districts as destinations for overnight trips from Choma District.

*Mobile phone ownership and travel differed by respondent groups in the travel survey:* Mobile phone ownership was high (69.4% in Choma, 79.2% in Ndola), with small differences in ownership among respondent age and caretaker status groups (see Table 1). In terms of travel, individuals residing in Choma were twice as likely to have taken an overnight trip in the last 2 months as those in Ndola (Choma: 19.5%; Ndola: 10.9%, *P* = 0.01). While travel by respondent group did not differ in Choma, in Ndola, caretakers were less likely to travel than other adults, although the sample sizes were small (see Table 1). Of reported trips, travel within a district was more common among individuals who did not own a mobile phone (83.7% vs 56.4% in Choma [*P* < .001] and 82.9% vs 53.0% in Ndola [*P* = .004]) across respondent groups. Across locations, adults were less likely to travel with children than caretakers (see Table 1). Further, individuals who owned a mobile phone were less likely to travel with children in Choma (21.9% vs 34.7%, *P* = 0.1). Finally, the average distance traveled varied by group and site; in Choma, mobile phone owners consistently traveled further than non-owners (see Figure 2, Table 1).

*Mobile phone data estimated more trips than the travel survey data:* The mobile phone data estimated more trips from each district and to a more diverse set of locations. On average, 8.1% of trips estimated from the mobile phone data were to other districts (minimum 0.4%, maximum 46.3%), with Lusaka, Kitwe, and Mpika as the most common travel destinations nationwide (see Figure 1B). When comparing these values to those estimated for Choma and Ndola Districts in the travel survey, the mobile phone data consistently estimated a higher proportion of out-of-district travel (Choma: 7.3% probability from mobile phone data vs 6.4% from the travel survey; Ndola: 6.6% from mobile phone data vs 3.3% from the travel survey). The mobile phone data also predicted more destination districts than the travel survey (see Figure 1C-D, Figure 2), likely because mobile phone data had a much larger number of “respondents” (subscribers) than the travel survey. Results were similar for estimates of travel by children. While mobile phone data do not allow for disaggregation of travel by age groups, assuming children traveled as often as adults would result in an estimate that 7.3% of children traveled out of Choma and 6.6% traveled out of Ndola. In comparison, from the travel survey, only 2.2% of children under 15 years of age traveled out of Ndola and 1.5% out of Choma. Adjusting the modeled probability of departure by results from the Choma travel survey resulted in a more dramatic reduction in probability than by results from the Ndola travel survey (Figure S2).

**Figure 3:**
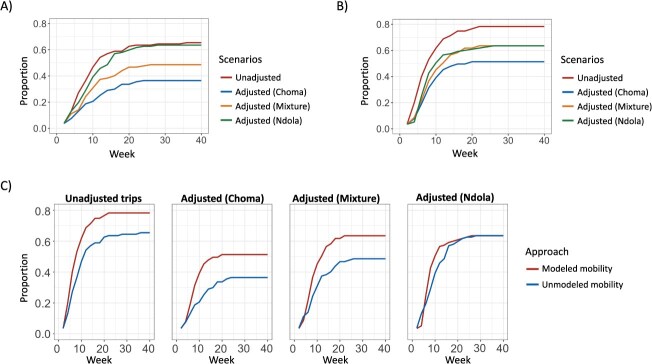
The proportion of districts with measles virus introduction events from modeled simulations where connectivity between districts was informed by Zamtel mobile phone data. (A) Proportion of districts with an introduction event, by week, with connectivity between districts informed by the probability of travel using mobile phone data. An introduction event is defined as having an upper confidence bound of cumulative infections greater than 1 case. (B) Proportion of districts with an introduction event, by week, with connectivity informed by the departure-diffusion model fitted to mobile phone data. (C) Proportion of districts with an introduction event, comparing approaches where mobile phone data were used with modeled mobility for each of the 4 adjustment scenarios.

**Figure 4:**
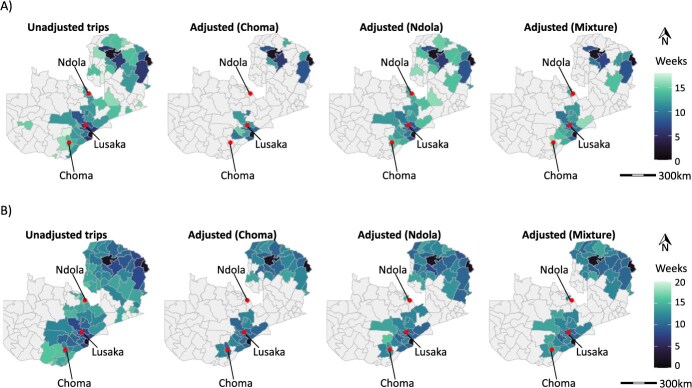
Results of measles virus transmission simulations using mobile phone data to estimate connectivity between districts. (A) Distribution of median time to introduction (weeks), with connectivity informed by the unadjusted and adjusted probability of travel using mobile phone data. The adjustment was made using results from the Choma travel survey, the Ndola travel survey, and a mixture of the Choma and Ndola travel surveys. (B) Distribution of median time to introduction (weeks), with connectivity informed by the unadjusted and adjusted probability of travel using the departure-diffusion model fitted to mobile phone data. The adjustment was done using results from the Choma travel survey, the Ndola travel survey, and a mixture of the Choma and Ndola travel surveys.

*Adjusting mobile phone data to account for children’s travel resulted in smaller outbreaks:* To investigate the impact of various connectivity measures on measles virus transmission, we integrated connectivity measures in a simulation model of measles across districts in Zambia. For analyses using only mobile phone data, the analysis was restricted to districts with mobility data (see Methods); when modeled mobility was used, all districts were included. Using unadjusted mobile phone data, outbreaks occurred in over half of the districts, with 70 districts experiencing measles virus introduction events by week 36. This number was reduced to 52 and 39 after adjusting to the results of the mixed Choma/Ndola and Choma travel surveys, respectively, and remained at 70 after adjusting to the Ndola travel survey (Figure 3A). The median time to the first introduced case also increased after adjustment, although this delay was minimal (about 1 week) (see Figure 4A). The adjustment resulted in fewer measles cases overall and in fewer mean numbers of cases in each province, except Northern and Lusaka Provinces, which were 2 of the 3 provinces where cases were seeded (see Table S3).

Modeled connectivity using a departure-diffusion model resulted in more districts with measles virus introduction events than for the simulations using mobile phone data alone (see Figure 3B-C). Population size, the 2020 SIA coverage, and distance from Lundazi District, which was initially seeded at the start of the simulation, were all positively associated with having measles virus introduction using the modeled mobility compared to the mobile phone data alone (see Table S5). The number of districts with an introduction event decreased from 78% when unadjusted mobile phone data were used to 51%, 63%, and 64% after adjusting to Choma, Ndola, and a mixture of Choma and Ndola travel survey results, respectively. Adjusting resulted in a slight delay in the median time to the introduction, although this delay was of short duration (0.4- to 0.9-week delay on average) (see Figure 4B). In all provinces except Lusaka, adjusting the estimated connectivity using the survey findings resulted in a lower incidence and a lower mean cumulative number of cases, except for the Southern Province (see Table S3). Locations with introductions only in the unadjusted approach were associated with a larger number of trips into districts and a further distance from Lundazi (see Table S5).

**Figure 5:**
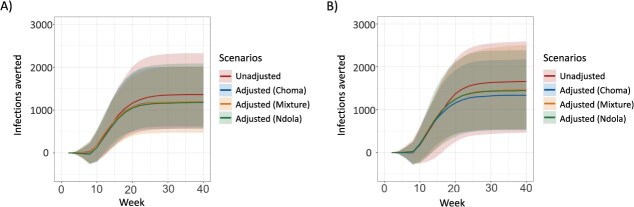
The estimated measles virus infections averted by the deployment of a district-wide reactive vaccination campaign. The solid line represents the median cumulative number of infections averted, with shaded 95% confidence intervals. (A) The vaccination campaign averted 46% to 47% of infections in all approaches, with connectivity between districts informed by the probability of travel using mobile phone data. (B) Using the departure-diffusion model, fitted to mobile phone data to quantify district connectivity. The vaccination campaign resulted in 47%, 48%, 50%, and 50% reduction in cumulative infections when connectivity was estimated by unadjusted mobile phone data, mobile phone data adjusted to results of the Choma travel survey, mobile phone data adjusted to the mixed the Choma and Ndola travel survey results, and mobile phone data adjusted to the Ndola travel survey, respectively.

*The relative effectiveness of a reactive vaccination campaign did not differ after adjusting to travel survey results:* In comparison to using the unadjusted mobile phone data, adjusting for travel by children based on the survey findings resulted in fewer districts reaching case thresholds to trigger a reactive vaccination campaign (48 districts in the unadjusted approach, compared with 17, 20, and 34 districts in adjusted approaches). However, the adjustment did not affect the relative change in cumulative infections following a vaccination campaign; in all scenarios considered, the reactive vaccination consistently resulted in a 46% to 47% reduction in the cumulative number of measles cases and a 10- to 12-week earlier end to the outbreak (see Table S6, Figure 5A). These results were consistent when using modeled mobility (see Table S6, Figure 5B). Sensitivity analysis for the effectiveness of a vaccination campaign in Lusaka, Northern, and Eastern Provinces showed similar results, although there was more variability in the percent reduction of cumulative infections with adjustment when using mobile phone data without fitting the departure-diffusion model (23%-30%) (see Figure S3, Table S6).

## Discussion

Estimating the risk of a measles outbreak is critical to effectively deploying additional vaccination strategies to achieve measles control and elimination. However, models to produce these estimates rely on data quantifying connectivity that may not capture the population of interest, such as travel by children. Using a travel survey, differences were identified between travel rates and the likelihood of traveling with a child by mobile phone ownership and respondent groups. Using a spatially dependent measles virus transmission model with connectivity values informed by mobile phone data adjusted for child travel using the survey findings resulted in fewer introduction events and delayed outbreaks across Zambia. However, the effectiveness of a reactive vaccination campaign did not differ by connectivity measure, although the number of targeted districts did. Specifically, mobile phone data and modeled mobility would result in more districts being recommended for additional vaccination campaigns, possibly diverting limited resources. While this may seem counterintuitive to the finding that the effectiveness of the campaign did not differ following adjustment, this is because adjustment of mobility measures resulted in a smaller overall outbreak size, even without the campaign.

The finding that children traveled less often than adults is consistent with existing literature across contexts, such as South Africa^32^ and the United States.^33^ For instance, children 0 to 6 years old in South Africa were between 1.6 and 3.3 times less likely to travel than adults 26 to 40 years old, depending on province. While to our knowledge, our study is the first to evaluate the impact of adjusting mobile phone data to better account for children’s travel, this heterogeneous travel by age and non-ubiquitous mobile phone ownership suggest that our findings are applicable in settings other than Zambia.

Nonetheless, the analysis has some key limitations. The mobile phone data were only analyzed from a single provider with varying coverage nationally, which resulted in some districts lacking data. Similarly, the travel survey was conducted in only 2 districts, and it is unclear to what extent these districts are representative of the country. Future work done in a more diverse set of districts could provide guidance on more accurate adjustments across locations.

Given a small proportion of individuals reported travel in the survey, the sample sizes were small, and caution should be taken when interpreting these results. Reporting of trips from the travel surveys could have also been affected by recall bias. Individuals may have been more likely to recall longer trips or those that were out of the ordinary; if shorter out-of-district trips were underreported, and children were more likely to accompany adults on these trips, then the estimates of children’s travel in the travel surveys would be underestimated. Reporting of travel may have also been affected if the responders were fatigued by the time the interview progressed to the travel module. This may have specifically resulted in reporting of smaller number of trips taken or only providing information about trips that were easier to remember.

Furthermore, although the questions asked in the travel survey have been used in previous surveys, it is possible that there was some variability in how the questions were asked by the data collectors, resulting in imperfect reliability.

The travel survey captured travel only during 2 months before the survey implementation. Unlike mobile phone data, this may have missed fluctuations in travel during high-peak travel times like holidays and cultural events, as well as seasonal mobility patterns. Furthermore, the travel survey was limited to individuals who were available at the time of the survey. Individuals who travel more are hence more likely to have been missed, which may result in underestimated probability of travel. The survey did collect data on whether household members stayed at the household the night before; individuals who did not were not eligible to be selected for the study. From these data, 15.5% of adults did not stay in the household the night before, compared with 2.9% of children under 5 years old. While this supports the likelihood that the travel survey underestimates travel by adults, it is unlikely that the magnitude of the underestimation of children’s travel in the survey was high.

In this study, we did not explore additional biases, such as socioeconomic status or occupation; however, these factors may further impact travel patterns as well as phone ownership. Finally, we made several simplifying assumptions to model measles virus transmission, such as focusing on spatial instead of age patterns (i.e., we did not use an age-specific contact matrix), which may mask finer-scale heterogeneity in outbreaks. In addition, we initiated our simulations in locations that first reported cases in 2021 and 2022, but routine surveillance may not accurately capture the true locations of measles virus introductions. However, sensitivity analyses, including introductions in other locations, such as the capital city of Lusaka (see Figure S4), were robust to these results. Additional work, including possible introductions along other high-risk areas such as international borders, should be considered in the future.

As countries aim to eliminate measles, evaluating tools such as transmission models to help guide policy decisions is vital. Accounting for biases in some national-level data, such as mobile phone data, based on detailed information from travel surveys can help refine estimates of connectivity. Especially as countries consider a shift toward a spatially targeted vaccination programs, understanding how and where these approaches may consistently classify subnational risk is important to tailor sub-national interventions.

## Supplementary Material

Web_Material_kwae304

## Data Availability

The individual travel survey data and mobile phone data underlying this article cannot be shared publicly due to confidentiality concerns. Under the National Health Research Act, the Government of Zambia does not allow public access to data collected in Zambia. All investigators interested in the data are required to submit a written request to the Ministry of Health. Contact the corresponding author to coordinate the request. Aggregated probabilities of travel are included in the supplementary materials. A simulated probability matrix from mobile phone data and district-level covariates used in malaria simulations are available in the GitHub repository (https://github.com/nkostandova/adjusting-mobility-for-children). Shapefiles for mapping are available at https://data.humdata.org/dataset/cod-ab-zmb.
