# Supplementary material for: Adjusting mobile phone data to account for children’s travel and the impact on measles dynamics in Zambia
Source: Am J Epidemiol. 2024 Aug 27;194(6):1584–94. doi: 10.1093/aje/kwae304 (PMC12133275; doi:10.1093/aje/kwae304)
Supplement: Web_Material_kwae304 [file web_material_kwae304.docx]

**Supplementary Material**

**Title:** Adjusting mobile phone data to account for children’s travel and the impact on measles dynamics in Zambia

**Authors:** Natalya Kostandova, Christine Prosperi, Simon Mutembo, Chola Nakazwe, Harriet Namukoko, Bertha Nachinga, Gershom Chongwe, Innocent Chilumba, Elliot N Kabalo, Kabondo Makungo, Kalumbu H Matakala, Gloria Musukwa, Mutinta Hamahuwa, Webster Mufwambi, Japhet Matoba, Irene Mutale, Edgar Simulundu, Phillimon Ndubani, Alvira Z Hasan, Shaun A Truelove, Amy K Winter, Andrea C Carcelen, Bryan Lau, William J Moss, Amy Wesolowski

**Table of Contents**

[Figure S1. Clusters selected for travel survey in Ndola and Choma districts, Zambia, 2022. 3](#_Toc168156931)

[Appendix S1. Summary of sampling strategy for the travel survey 4](#_Toc168156932)

[Table S1. Profile of individuals enrolled in the travel survey 4](#_Toc168156933)

[Appendix S2. Estimation of trips taken by children using travel survey results in Choma and Ndola Districts 5](#_Toc168156934)

[Appendix S3. Model fit diagnostics for gravity, radiation, and departure-diffusion model fit to unweighted call data records 8](#_Toc168156935)

[Table S2. Parameters of departure-diffusion model, fitted to call data records 12](#_Toc168156936)

[Appendix S4. Transmission model specification 12](#_Toc168156937)

[Appendix S5. Graphical summary of methods 17](#_Toc168156938)

[Figure S2. Probability of departure from Choma and Ndola districts in the unadusted approach and after adjusting using results from Choma travel survey (“Adjusted (Choma)”), Ndola travel survey (“Adjusted (Ndola)”), and mixture of the Choma and Ndola travel surveys (“Adjusted (Mixture)”). 21](#_Toc168156939)

[Table S3. Mean cumulative infections and change in cumulative infections after adjusting the probability of travel using results from travel survey (Choma and Ndola districts) 22](#_Toc168156940)

[Table S4. Results of multivariable analysis of district characteristics associated with district having at least 10% introduction probability in the approach using departure-diffusion model only, and not in the approach directly using mobile phone data to quantify district connectivity 23](#_Toc168156941)

[Table S5. Results of multivariable analysis of district characteristics associated with district having at least 10% introduction probability only in the unadjusted approach. 23](#_Toc168156942)

[Table S6. Median duration of outbreak and cumulative infections under no reactive vaccination campaign and reactive vaccination campaign scenarios. 25](#_Toc168156943)

[Figure S3. Estimated measles infections averted by the deployment of a province-level supplementary immunization activity (SIA). 28](#_Toc168156944)

[Figure S4. Sensitivity analysis, with the initial introduction of cases in Lusaka district only. 29](#_Toc168156945)

## Figure S1. Clusters selected for travel survey in Ndola and Choma districts, Zambia, 2022.

## Appendix S1. Summary of sampling strategy for the travel survey

In both districts, 36 clusters were selected from established Standardized Enumeration Areas using probability proportional to population size (see Figure S1). Within each selected cluster, households were enumerated and three separate samples of households were selected: a) households with children 1 – 4 years of age; b) households with children 5 – 14 years of age; and c) households with an adult (all listed households). For each household selected, if more than one individual was eligible to participate (e.g. more than one individual in group of interest), the participant was selected at random. In addition, individuals could be enrolled in multiple eligible groups, with the largest overlaps between the two caregiver groups (52% of younger children caregivers, 27% of older children caregivers, see Table *S1*).

## Table S1. Profile of individuals enrolled in the travel survey

|  | **Choma** | **Ndola** |
| --- | --- | --- |
| **Individuals enrolled** |  |  |
| Caregivers of children 1 – 4 years old | 444 | 321 |
| Caregivers of children 5 – 14 years old | 835 | 631 |
| Adults 15 years and older | 777 | 569 |
| **Individuals responding for multiple categories in the same household** |  |  |
| Same respondent for caregivers of children 1 – 4 and 5 – 14 | 220 | 133 |
| Same respondent for caregivers of children 5 – 14 and adult | 190 | 91 |
| Same respondent for caregivers of children 1 – 4 and adult | 118 | 50 |
| Same respondent for all three categories | 36 | 9 |

## Appendix S2. Estimation of trips taken by children using travel survey results in Choma and Ndola Districts

*Choma:*


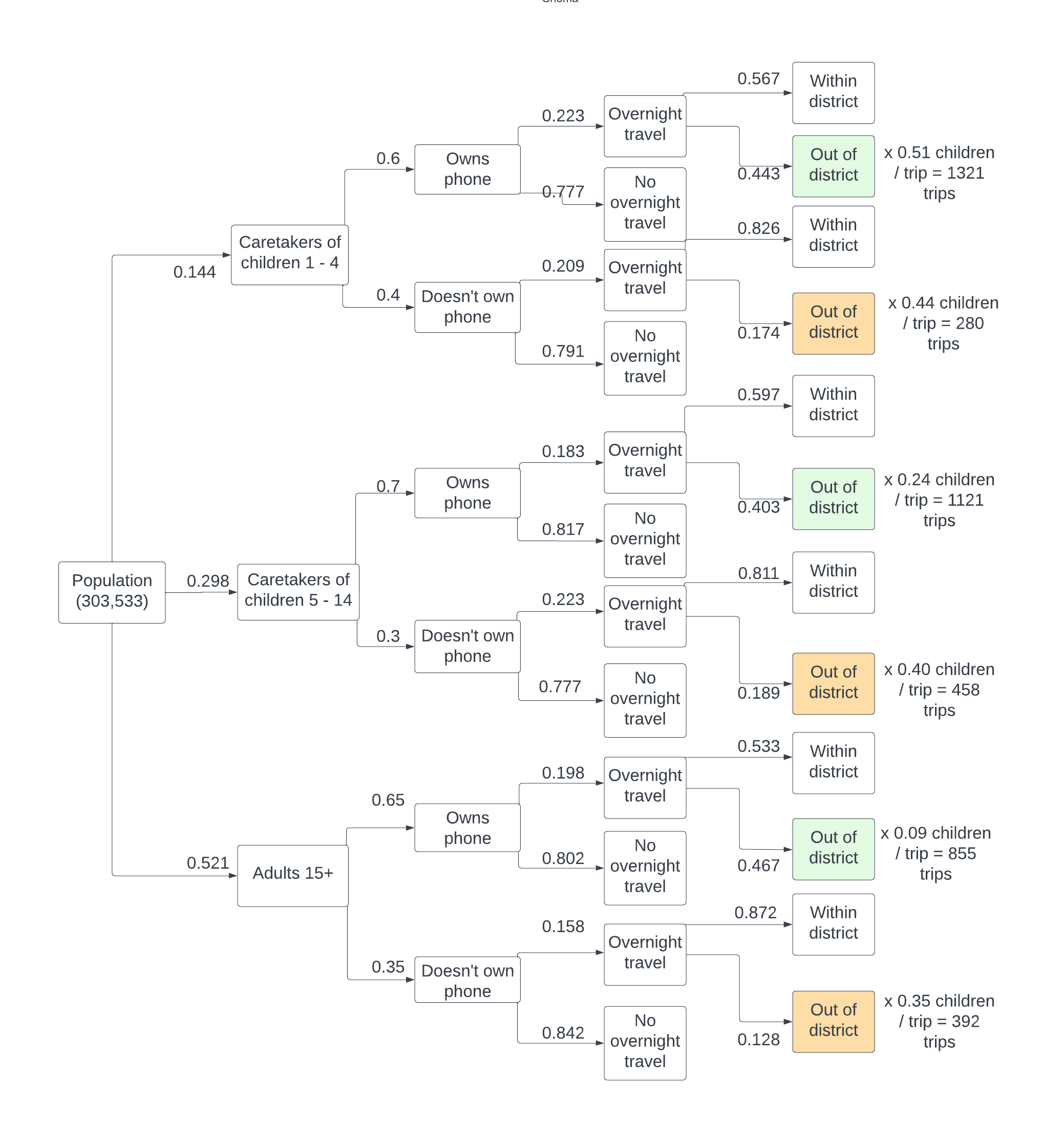


Total number of overnight trips outside district estimated to have been taken by children over two months is 4,427.

Over 1 month, this corresponds to ~1.52% of children

In mobile phone data, in Choma, 7.3% of trips were outside of district

*Ndola:*


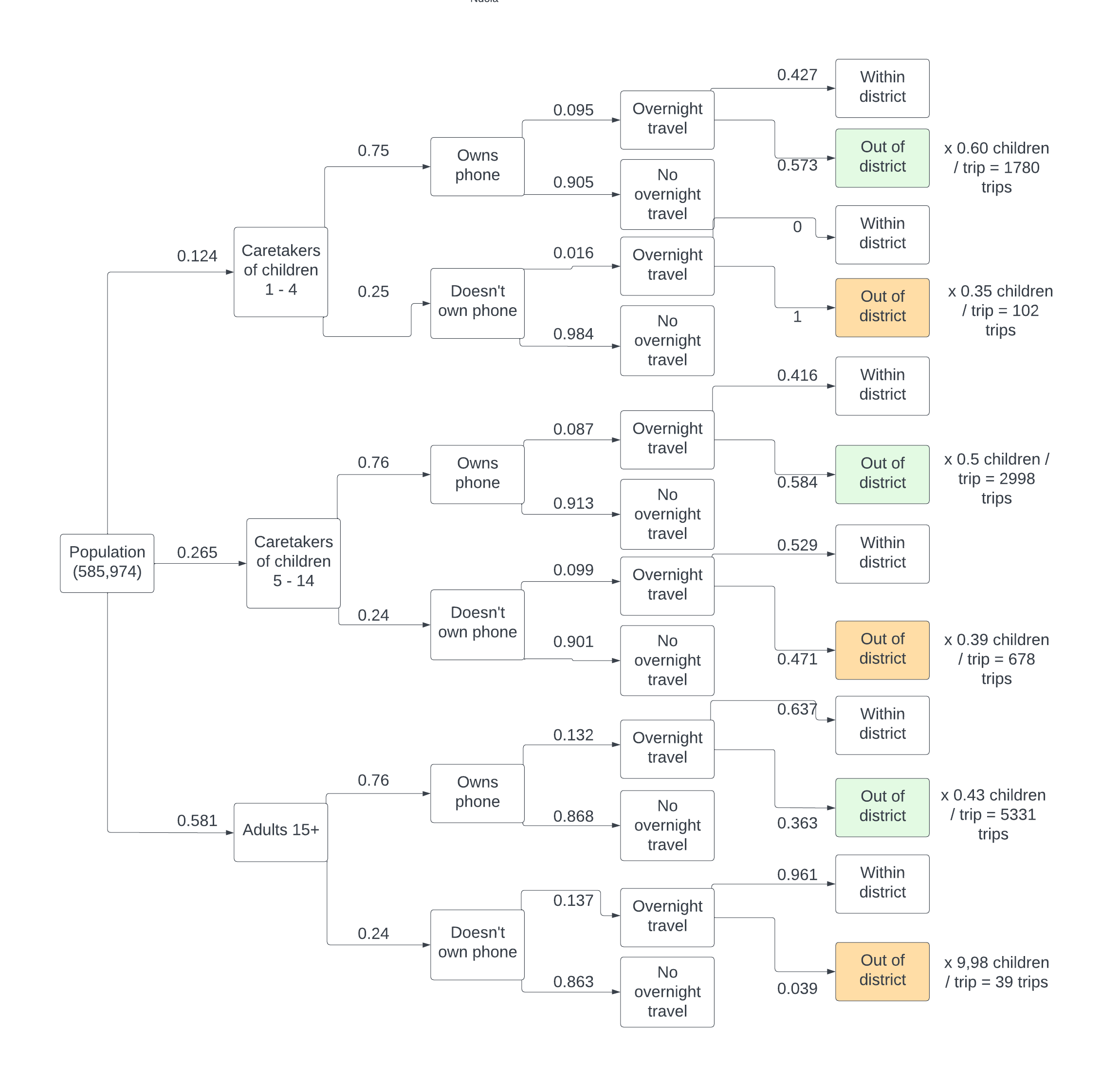
 Total number of overnight trips outside district estimated to have been taken by children over two months is 10,928 trips with children.

Over 1 month, this corresponds to ~2.22% of children

In mobile phone data, in Ndola, 6.6% of trips were outside of district

## Appendix S3. Model fit diagnostics for gravity, radiation, and departure-diffusion model fit to unweighted call data records

| Model family | Model | DIC^1^ | RMSE^2^ | MAPE^3^ | R2 |
| --- | --- | --- | --- | --- | --- |
| Gravity | Basic | 459496755 | 35,414,396.58 | 3,714,190,722.35 | 0.5000080 |
|  | Transport | 63985751 | 15,390.68 | 280,882.35 | 0.2005823 |
|  | Power | 63958048 | 15,494.40 | 273,007.96 | 0.2107881 |
|  | Exponential | 28207334 | 16,779.59 | 319,827.75 | 0.3952551 |
|  | Normalized power law | 64154754 | 14,865.41 | 269,853.46 | 0.1630555 |
|  | Normalized exponential | 31341997 | 15,740.58 | 335,976.53 | 0.3292897 |
| Radiation | Basic | NA | 16,968.22 | 590,831.45 | 0.5488763 |
|  | Finite | NA | 18,492.76 | 617,965.11 | 0.5497407 |
| Departure diffusion | Exponential gravity | 76328739 | 172,445.36 | 23,473.45 | 0.7952939 |
|  | Power gravity | 76126750 | 172,468.16 | 54,579.43 | 0.7953547 |
|  | Radiation | 82113597 | 172,538.56 | 15,964.05 | 0.7951058 |

^1^DIC: Deviance information criterion

^2^RMSE: Root mean square error

^3^MAPE: Mean absolute percentage error

In all these models, the number of trips from origin $i$ to destination $j$ follows a Poisson distribution with mean $\lambda_{ij}$. The formulations for gravity models for $\lambda_{ij}$ are presented below, where $N_{i}$ is population size at origin, $N_{j}$ is population size at destination, and $d_{ij}$ is the distance between origin and destination.

| Model family | Model | Formulation |
| --- | --- | --- |
| Gravity | Basic | $\lambda_{ij}=\theta\left( \frac{N_{i}N_{j}}{d_{ij}} \right)$ |
|  | Transport | $\lambda_{ij}=\theta\left( \frac{N_{i}N_{j}}{d_{ij}^{\gamma}} \right)$ |
|  | Power | $\lambda_{ij}=\theta\left( \frac{N_{i}^{\omega_{1}}N_{j}^{\omega_{2}}}{d_{ij}^{\gamma}} \right)$ |
|  | Exponential | $\lambda_{ij}=\theta\left( \frac{N_{i}^{\omega_{1}}N_{j}^{\omega_{2}}}{e^{d_{ij}/\delta}} \right)$ |
|  | Normalized power law | $\lambda_{ij}=\theta N_{i}\left( \frac{N_{j}^{\omega}d_{ij}^{-\gamma}}{\sum_{j} N_{j}^{\omega}d_{ij}^{-\gamma}} \right)$ |
|  | Normalized exponential | $\lambda_{ij}=\theta N_{i}\left( \frac{N_{j}^{\omega}e^{{-d}_{ij}/\delta}}{\sum_{j} N_{j}^{\omega}e^{{-d}_{ij}/\delta}} \right)$ |

Radiation models are a parameter-free approach, with total number of trips from origin $i$ to destination $j$ inversely proportional to the total population within the circle with radius $d_{ij}$. In formulations below, $s_{ij}$ is the total population residing within radius $d_{ij}$ from origin $i$. $M_{i}$ is the number of trips originating from $i$ to all destinations.

| Model family | Model | Formulation |
| --- | --- | --- |
| Radiation | Basic | $\lambda_{ij}=M_{i}\frac{N_{i}N_{j}}{(N_{i}+s_{ij})(N_{i}+N_{j}+s_{ij})}$ |
|  | Finite | $\lambda_{ij}=\frac{M_{i}}{1-N_{i}/\sum_{i} N_{i}}\frac{N_{i}N_{j}}{(N_{i}+s_{ij})(N_{i}+N_{j}+s_{ij})}$ |

Finally, in departure-diffusion models, for the departure component, the probability of travel outside origin $i$, $\tau_{i}$, is estimated hierarchically, with binomial probabilities for each origin $i$ drawn from a Beta distribution, with parameters estimated as population-level hyper-priors. The diffusion process for the conditional probability of travel from origin $i$ to destination $j$ is modelled independently from departure. The formulations for the diffusion process is as follows:

| Model family | Model | Formulation |
| --- | --- | --- |
| Departure diffusion | Exponential gravity | $\pi_{ij}=\frac{N_{j}^{\omega}e^{{-d}_{ij}/\delta}}{\sum_{\forall j\neq i} N_{j}^{\omega}e^{{-d}_{ij}/\delta}}$ |
|  | Power gravity | $\pi_{ij}=\frac{N_{j}^{\omega}d_{ij}^{-\gamma}}{\sum_{\forall j\neq i} N_{j}^{\omega}d_{ij}^{-\gamma}}$ |
|  | Radiation | $\pi_{ij}=\frac{\frac{N_{j}}{(N_{i}+s_{ij})(N_{i}+N_{j}+s_{ij})}}{\sum_{\forall j\neq i} \frac{N_{j}}{(N_{i}+s_{ij})(N_{i}+N_{j}+s_{ij})}}$ |

More details about the models are provided elsewhere (<https://covid-19-mobility-data-network.github.io/mobility/articles/V5_list_models.html>).

## Table S2. Parameters of departure-diffusion model, fitted to call data records

| Parameter | Mean | SD |
| --- | --- | --- |
| $\delta$ | 59388.36 | 15.5 |
| $\theta$ | 0.91198 | 0.00028 |
| $\tau$ | 0.06560 | 0.00002 |
| $\theta$ | 11.9742 | 0.00085 |

## Appendix S4. Transmission model specification

We used a spatial Maternal Immunity-Susceptible-Infected-Vaccinated-Removed discrete compartmental stochastic model to simulate measles transmission dynamics (Figure 1). Time steps were set to two weeks, which correspond to generation time. Given high vaccination coverage in Zambia, we assume that all newborns enter a maternal immunity compartment, where they are not at risk for measles infection. Individuals then transition from maternal immunity (M) to susceptible (S), with the remaining compartments following a standard SIR model (infected (I), and removed (R) compartments). Individuals that receive first dose of vaccination via campaign or routine immunization activities either become immune (move to R) or do not develop immunity and remain susceptible to infection (V1C for those that received first dose via campaign or V1R for those that received first dose via routine activities). Receiving second dose vaccine is assumed to be immunizing as individuals move to V2 compartment.


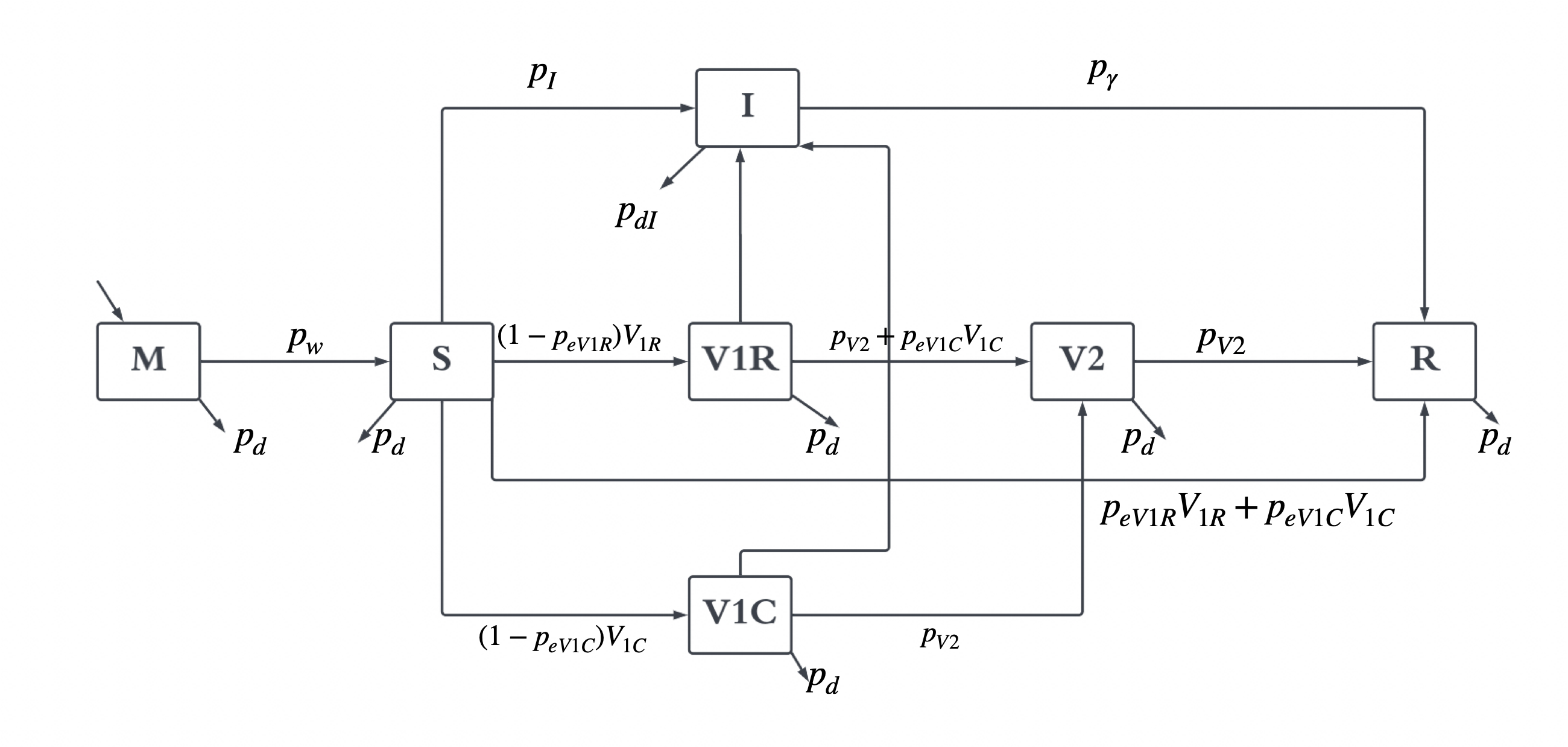


Figure 1. Compartmental model for measles transmission.

At beginning of each time step, we simulated population movement. For each district *i*, for each compartment, we carried out a multinomial draw, where an individual had a probability of moving to district *j* based on mobility matrix as described above.

Next, we set up a transition matrix $T$, where each element $T_{AB}$ represents the probability of movement from compartment $A$ to $B$ at each discrete time step. These probabilities are presented in Table 1.

Table 1. Transitions between compartments in transmission model

| Event / Transition | Description | Probability of transition | Formula | Parameter value |
| --- | --- | --- | --- | --- |
| $M\to S$ | Waning of maternal immunity | $p_{w}$ | $1-exp(-\omega\Delta t)$ | $\omega$ = rate of waning immunity; 0.45 |
| $M\to death$  $S\to death$  $V1R\to death$  $V1C\to death$  $V2\to death$  $R\to death$ | Death due to non-measles causes (background death) | $p_{d}$ | $1-exp(-\mu\Delta t)$ | $\mu$ = rate of death; calculated as province-level crude mortality rate, divided by 1,000, divided by 26 |
| $I\to death$ | Death due to measles or other causes for infected individuals | $p_{dI}$ | $1-exp(-\mu\Delta t-\mu_{I}\Delta t)$ | $\mu$ = rate of background death as above; $\mu_{I}$ = rate of death from measles, taken to be 0.0013(1) |
| $S\to I$ | Infection of susceptible individuals | $p_{I}$ | $1-exp(-\frac{\beta_{t}I^{\alpha}}{N}dw \Delta t)$ | $\beta_{t}$ is a seasonal transmission coefficient; $\beta_{t}= \beta_{0}(1+\beta_{1}cos(2\pi t)$; $\alpha$ is mixing parameter = 0.975(2) $dw$ = a random draw from a truncated normal distribution with mean =1 and sd = 0.01 |
| $I\to R$ | Recovery after infection | $p_{\gamma}$ | $1-exp(-\gamma\Delta t)$ | $\gamma$ is recovery rate; given that time between time steps is approximately equal to infectious period (~14 days), $\gamma=1$. |
| $S\to R$ | Immunization of susceptible individuals; occurs if individual receives an effective first dose of vaccine (either through routine or through SIA) | $p_{eV1R}V_{1R}+p_{eV1C}V_{1C}$ | $V_{1R}=1-exp(-\frac{{MCV1}_{t}}{MCV1rate}\Delta t$)  $V_{1C}=1-exp(-{SIA}_{t}\Delta t$) | $p_{eV1R}$ is effectiveness of first routine dose of vaccination through routine services; taken to be 85%.  $p_{eV1C}$ is effectiveness of first routine dose of vaccination through routine services; taken to be 93%. $MCV1rate$ is rate of receiving first dose of vaccine through routine; set to (9 months – 1 / rate of waning immunity) ~ about 13 weeks |
| $S\to V1R$ | Moving to compartment V1R after receiving a non-effective dose of MCV1 through routine | $(1-p_{eV1R})V_{1R}$ | As above | As above |
| $S\to V1C$ | Moving to compartment V1R after receiving a non-effective dose of MCV1 through campaign | $(1-p_{eV1C})V_{1C}$ | As above | As above |
| $V1R\to V2$ | Receiving second dose of vaccination (either second routine dose, or via campaign) | $p_{V2}$ $+p_{eV1C}V_{1C}$ | $p_{V2}=1-exp(-\frac{{MCV2}_{t}}{MCV2rate}\Delta t$) | $p_{eV1C}V_{1C}$ as described above  ${MCV2}_{t}$ = coverage of MCV2  $MCV2rate$ = 9 months (18 bi-weeks), taken as average time between first and second dose of MCV. Assume 100% MCV2 effectiveness. |
| $V1C\to V2$ | Receiving second dose of vaccination via routine dose | $p_{V2}$ | $p_{V2}=1-exp(-\frac{{MCV2}_{t}}{MCV2rate}\Delta t$) | As above |
| $V2\to R$ | Movement from second dose compartment to recovered | $p_{\partial}$ |  | Probability equals to 1. V2 and R are functionally the same; V2 set up primarily to keep track of number of individuals receiving second dose of MCV. |

Birth and mortality rates were obtained from province-level administrative data. We assumed that all births go into the maternal immunity compartment. We assumed that mortality rates are the same across compartments, with exception of higher mortality rates for infected individuals.

Population data: District-level population size was obtained from official 2011-2035 projections based on the 2010 Zambia census (3). The initial population immune to measles due to the presence of maternally-acquired antibodies was estimated as the number of children younger than 3 months calculated as number of children younger than 5 years divided by 20.

Population-level vaccination coverage and measles seropositivity: District-level measles vaccination data for 115 districts in 2018 was obtained from Zambia’s Expanded Program on Immunization program via the Zambia Health Management Information System. From 2019 onwards, because administrative boundaries changed, we obtained district-level MCV1 coverage by scaling district-level 2018 coverage proportional to changes in national coverage in succeeding years. MCV2 coverage was estimated by scaling MCV1 proportional to the ratio between national MCV2 and MCV1 coverage (WHO and UNICEF 2022). All coverage estimates were capped at 99%. 2016 and 2020 SIA coverage estimates were obtained from Post-Coverage Evaluation Survey reports (4,5). While these were province-level, we assumed districts within the province had the same coverage. We set the initial susceptible population based on population-level susceptibility estimates from a national serological survey conducted in 2016 across districts in Zambia (6). Specifically, for each simulation, district-level proportions immune were drawn from a random distribution with bounds specified in the serological survey. The number of individuals susceptible was obtained by multiplying the complement of this value by the estimated population in the district.

At each step $t+1$, individuals in compartment $A$ transition to compartments in the model according to multinomial draw.

## Appendix S5. Graphical summary of methods


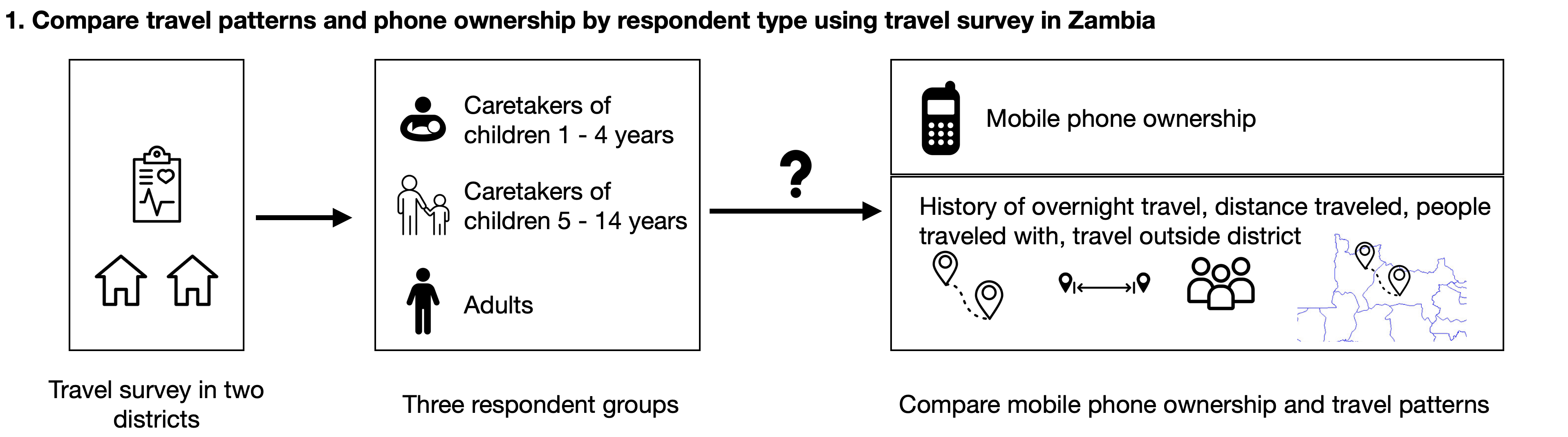


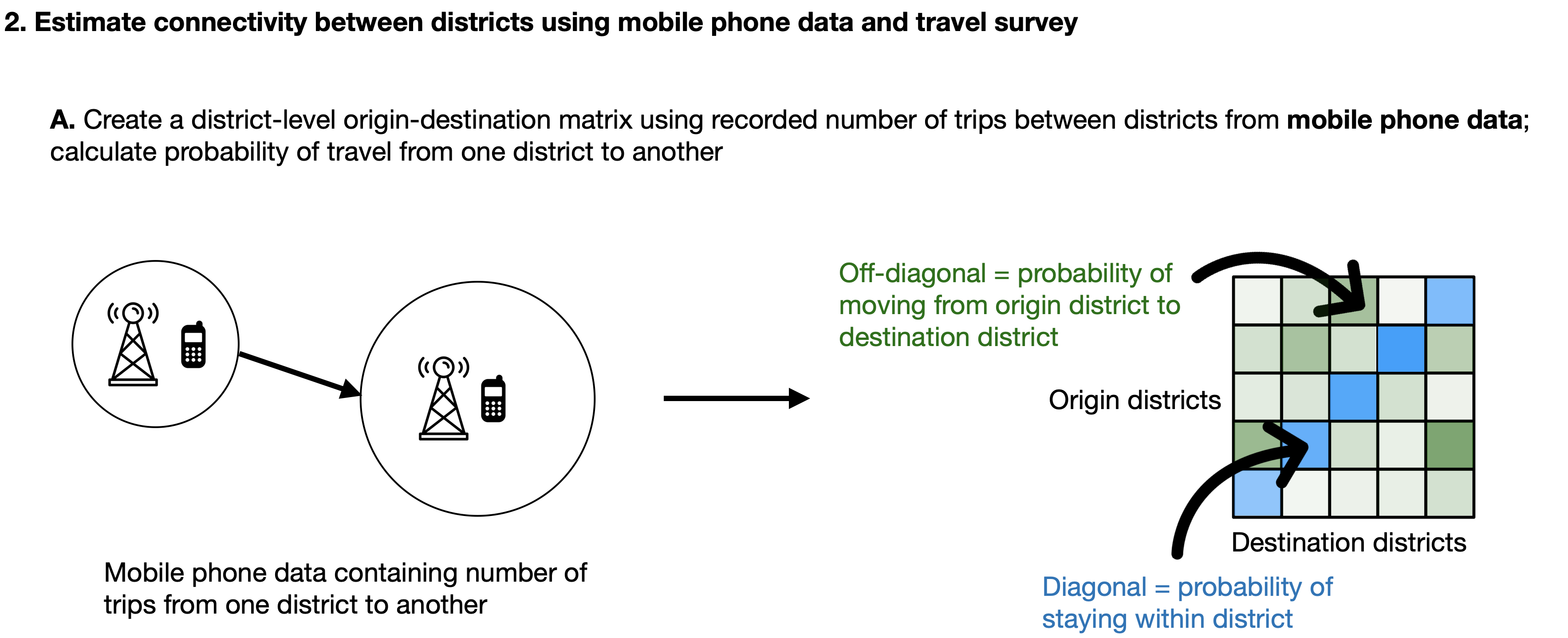


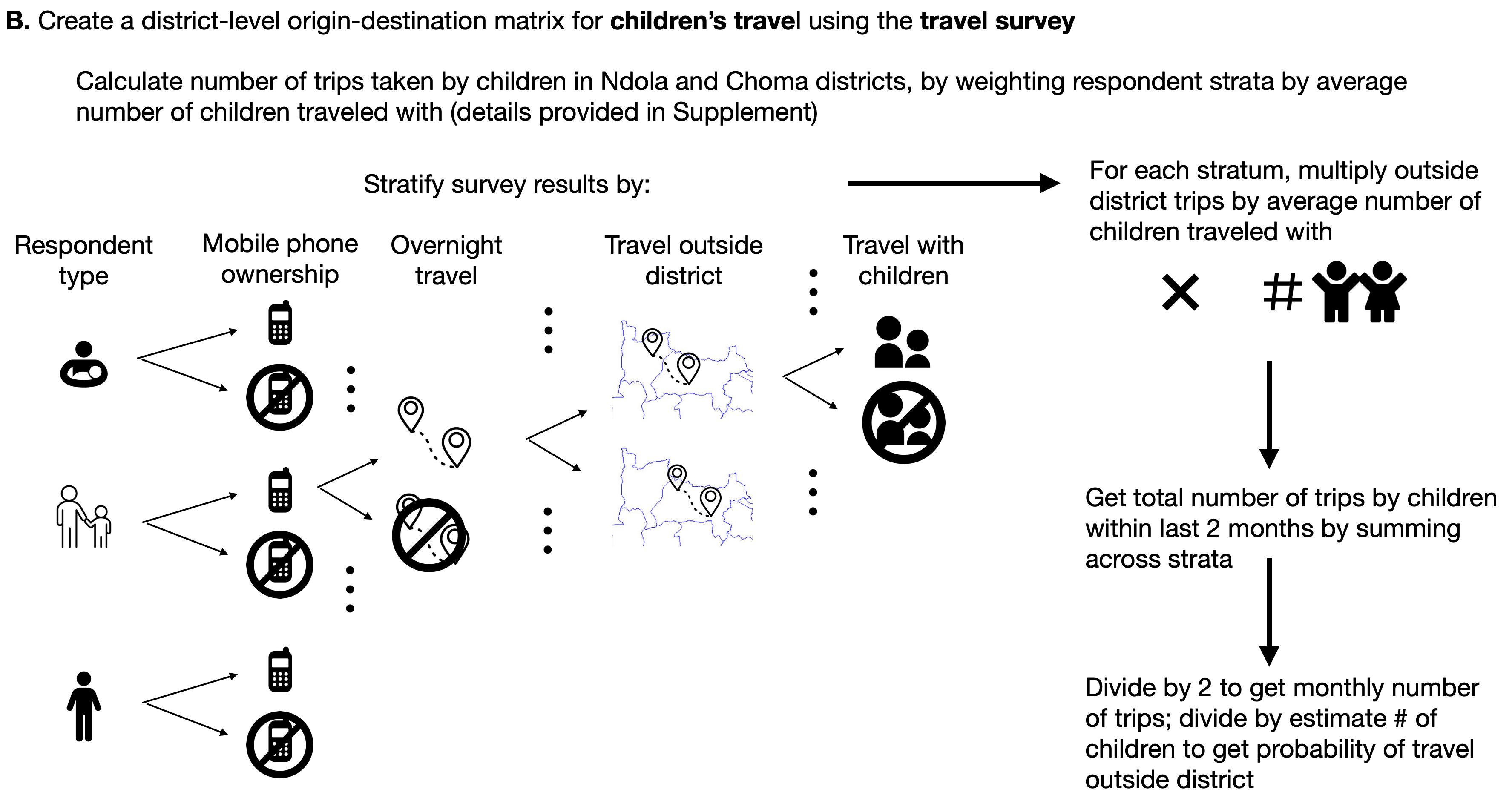


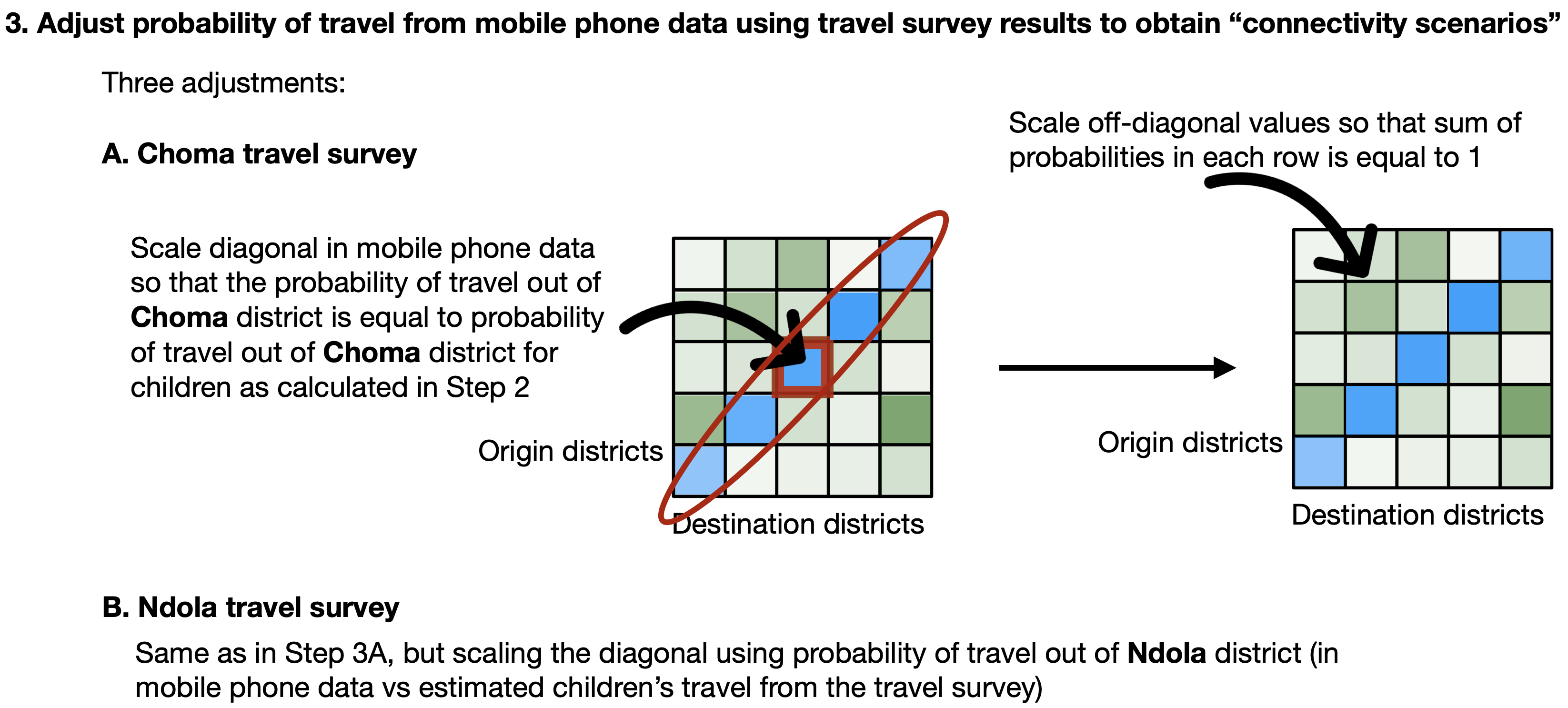


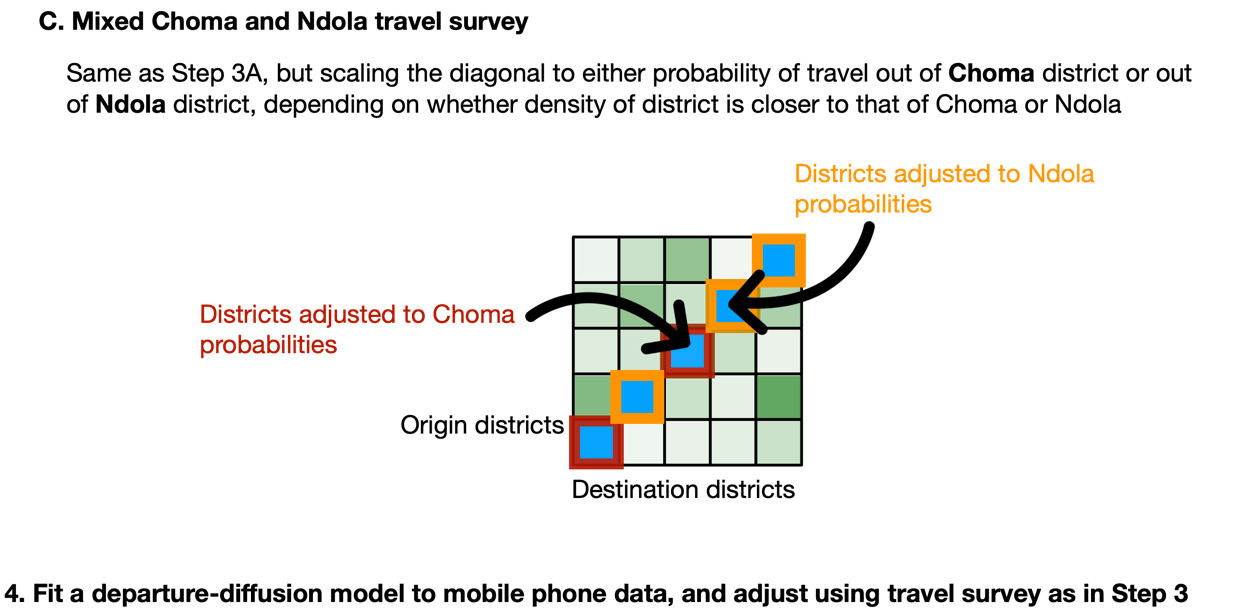


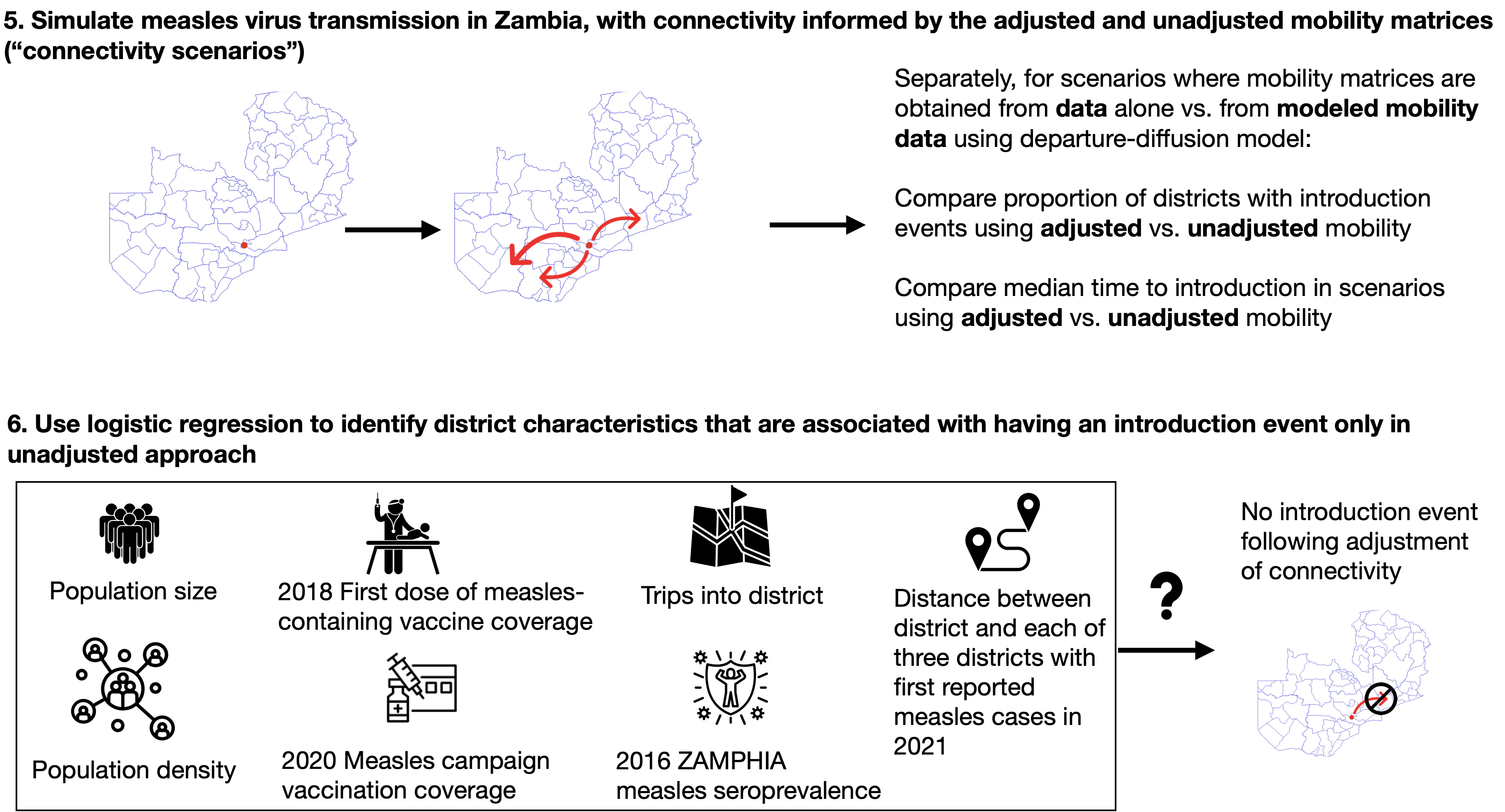


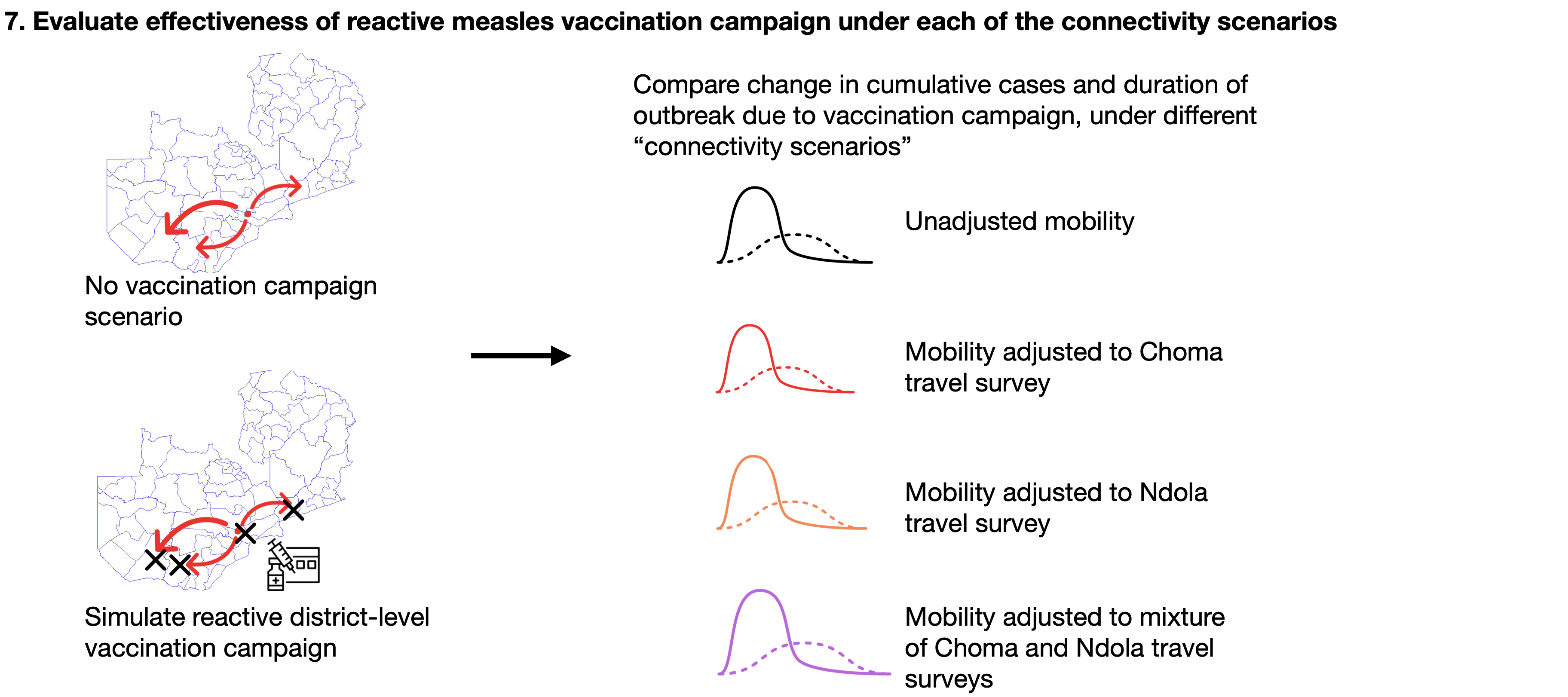


## Figure S2. Probability of departure from Choma and Ndola districts in the unadusted approach and after adjusting using results from Choma travel survey (“Adjusted (Choma)”), Ndola travel survey (“Adjusted (Ndola)”), and mixture of the Choma and Ndola travel surveys (“Adjusted (Mixture)”).


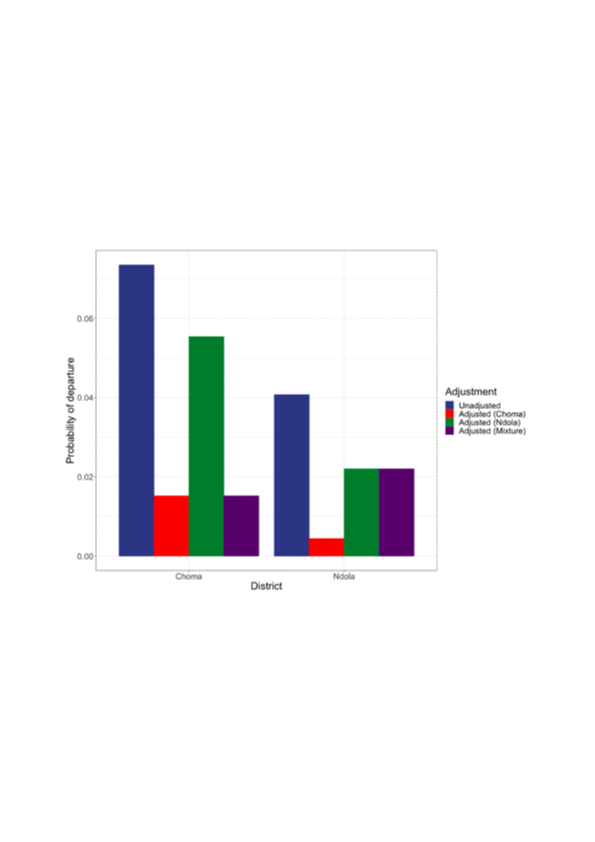


## Table S3. Mean cumulative infections and change in cumulative infections after adjusting the probability of travel using results from travel survey (Choma and Ndola districts)

| Province | Raw mobile phone data | | | | Departure-diffusion model | | |  |
| --- | --- | --- | --- | --- | --- | --- | --- | --- |
|  | Unadjusted cumulative infections (mean) | % Change when adjusted by Choma travel survey | % Change when adjusted by Ndola travel survey | % Change when adjusted by mixture travel survey | Unadjusted cumulative infections (mean) | % Change when adjusted by Choma travel survey | % Change when adjusted by Ndola travel survey | % Change when adjusted by mixture travel survey |
| Central | 67 | -90 | -44 | -52 | 104 | -72 | -64 | -63 |
| Copperbelt | 27 | -94 | -40 | -57 | 16 | -88 | -62 | -81 |
| Eastern | 11 | -95 | -55 | -81 | 38 | -87 | -63 | -82 |
| Luapula | 6 | -90 | 11 | -35 | 101 | -84 | -54 | -66 |
| Lusaka | 1,434 | -6 | -5 | -6 | 1,459 | -1 | 0 | 1 |
| Muchinga | 812 | -13 | -13 | -14 | 836 | -14 | -7 | -10 |
| North-Western | 1 | -95 | -81 | 35 | 1 | -100 | -100 | -100 |
| Northern | 461 | -5 | 0 | -6 | 655 | -22 | -20 | -22 |
| Southern | 95 | -72 | -22 | -69 | 185 | -74 | -67 | -53 |
| Western | 1 | -22 | -45 | -85 | 0 | NA | NA | NA |
| **National (N, % change)** | **2,916** | **2,532**  **(-13%)** | **2,661**  **(-9%)** | **2,571**  **(-12%)** | **3,395** | **2,784**  **(-18%)** | **2,918**  **(-14%)** | **2,907**  **(-14%)** |

Table S4. Results of multivariable analysis of district characteristics associated with district having at least 10% introduction probability in the approach using departure-diffusion model only, and not in the approach directly using mobile phone data to quantify district connectivity**.**

| Variable |  | OR | 2.5 % | 97.5 % | p |
| --- | --- | --- | --- | --- | --- |
| 2020 population (thousands) |  | 1.040 | 1.008 | 1.083 | **0.029** |
| PCES 2020 coverage |  | 1.222 | 1.064 | 1.474 | **0.013** |
| ZAMPHIA seropositivity |  | 1.101 | 0.882 | 1.396 | 0.395 |
| Density |  | 0.965 | 0.898 | 0.990 | 0.224 |
| MCV1 2018 coverage |  | 1.025 | 0.962 | 1.121 | 0.489 |
| Trips in (10,000) |  | 0.426 | 0.183 | 0.770 | **0.020** |
| Distance from Chirundu (km) |  | 1.000 | 0.994 | 1.006 | 0.993 |
| Distance from Lunte (km) |  | 0.999 | 0.991 | 1.005 | 0.691 |
| Distance from Lundazi (km) |  | 1.007 | 1.002 | 1.014 | **0.024** |

Table S5. Results of multivariable analysis of district characteristics associated with district having at least 10% introduction probability only in the unadjusted approach.

| Variable |  | Mobile phone data (unfitted) | | | | Mobile phone data (fitted departure-diffusion model) | | | |  |
| --- | --- | --- | --- | --- | --- | --- | --- | --- | --- | --- |
|  |  | OR | 2.5 % | 97.5 % | p | OR | 2.5 % | 97.5 % | p |  |
| 2020 population (1,000)^1^ |  | 1.016 | 1.001 | 1.035 | 0.051 | 1.027 | 1.008 | 1.054 | **0.016** |  |
| PCES 2020 coverage^2^ |  | 1.203 | 1.016 | 1.509 | 0.057 | 1.161 | 1.028 | 1.372 | **0.037** |  |
| ZAMPHIA seropositivity^3^ |  | 0.684 | 0.420 | 0.979 | 0.071 | 0.794 | 0.542 | 1.076 | 0.185 |  |
| Density^4^ |  | 0.984 | 0.964 | 0.996 | 0.070 | 0.996 |  | 1.000 | 0.134 |  |
| MCV1 2018 coverage^5^ |  | 0.991 | 0.927 | 1.064 | 0.801 | 1.000 | 0.943 | 1.055 | 0.987 |  |
| Trips in (10,000)^6^ |  | 0.823 | 0.674 | 0.956 | **0.026** | 0.647 | 0.452 | 0.837 | **0.004** |  |
| Distance from Chirundu (km)^7^ |  | 0.999 | 0.992 | 1.007 | 0.890 | 0.999 | 0.992 | 1.006 | 0.768 |  |
| Distance from Lunte (km)^7^ |  | 0.996 | 0.987 | 1.004 | 0.321 | 0.996 | 0.988 | 1.003 | 0.307 |  |
| Distance from Lundazi (km)^7^ |  | 1.008 | 1.001 | 1.016 | **0.038** | 1.006 | 1.001 | 1.012 | **0.036** |  |

^1^Estimated 2020 population (in thousands) in each district, as projected using results of 2010 census (3)

^2^Estimated coverage from 2020 supplementary immunization activity (province-level), in percent

^3^ Mean district-level measles seropositivity, from 2016 ZAMPHIA biorepository (6)

^4^District-level density, from 2010 census projections (3)

^5^Estimated 2018 MCV1 coverage, as per administrative estimates.

^6^Inbound trips into district, estimated as a sum of all trips into district from other districts from Zamtel mobile phone data, in 10,000

^7^Distance from centroid of district to centroid of Chirundu, Lunte, or Lundazi districts. These three districts are where the initial infectious cases were seeded.

Table S6. Median duration of outbreak and cumulative infections under no reactive vaccination campaign and reactive vaccination campaign scenarios. **Two vaccination campaign strategies are considered – district-level reactive vaccination, wherein a campaign is triggered in each district that reaches 3 cumulative infections, and province-level vaccination, wherein vaccination is carried out in three provinces with initial infections.**

| Scenario | Median duration of outbreak, no vaccination campaign (weeks) | Median duration of outbreak, with vaccination campaign (weeks) | Cumulative Infections, no vaccination campaign (median, 95% CI) | Cumulative Infections, with vaccination campaign (median, 95% CI) | Median percent reduction in cumulative infections following vaccination campaign |
| --- | --- | --- | --- | --- | --- |
| *District-level reactive vaccination campaign: using mobile phone data* | | | | | |
| Unadjusted mobile phone data | 40 | 28 | 2879 [2146; 3927] | 1516 [1144; 2050] | 47 |
| Adjusted (Choma) | 36 | 26 | 2482 [1842; 3415] | 1307 [945; 1744] | 47 |
| Adjusted (Mixed) | 36 | 26 | 2539 [1933; 3422] | 1340 [1010; 1742] | 47 |
| Adjusted (Ndola) | 38 | 28 | 2619 [1951; 3564] | 1410 [1006; 1896] | 46 |
| *District-level reactive vaccination campaign: using departure-diffusion model fit to mobile phone data* | | | | | |
| Unadjusted mobile phone data | 46 | 30 | 3364 [2291; 4635] | 1780 [1250; 2359] | 47 |
| Adjusted (Choma) | 38 | 26 | 2712 [1920; 3799] | 1402 [1065; 1824] | 48 |
| Adjusted (Mixed) | 40 | 26 | 2802 [1974; 4350] | 1392 [1019; 1822] | 50 |
| Adjusted (Ndola) | 42 | 28 | 2873 [1935; 4147] | 1431 [1078; 1903] | 50 |
| *Province-level vaccination campaign (Lusaka, Northern, Eastern provinces): using mobile phone data* | | | | | |
| Unadjusted mobile phone data | 40 | 34 | 2879 [2146; 3927] | 2012 [1364; 2847] | 30 |
| Adjusted (Choma) | 36 | 32 | 2482 [1842; 3415] | 1832 [1320; 2487] | 26 |
| Adjusted (Mixed) | 36 | 32 | 2539 [1933; 3422] | 1832 [1310; 2550] | 28 |
| Adjusted (Ndola) | 38 | 32 | 2619 [1951; 3564] | 1907 [1360; 2735] | 27 |
| *Province-level vaccination campaign (Lusaka, Northern, Eastern provinces): using departure-diffusion model fit to mobile phone data* | | | | | |
| Unadjusted mobile phone data | 46 | 34 | 3364 [2291; 4635] | 2302 [1699; 3327] | 32 |
| Adjusted (Choma) | 38 | 34 | 2712 [1920; 3799] | 1938 [1364; 2822] | 29 |
| Adjusted (Mixed) | 40 | 36 | 2802 [1974; 4350] | 1981 [1329; 2935] | 29 |
| Adjusted (Ndola) | 42 | 36 | 2873 [1935; 4147] | 2007 [1422; 3170] | 30 |

Figure S3. Estimated measles infections averted by the deployment of a province-level supplementary immunization activity (SIA). **SIA is carried out in the three provinces where the first cases of measles were reported in 2021-2022 (Northern, Lusaka, and Eastern provinces).** The solid line represents the median cumulative number of infections averted, with shaded 95% confidence intervals. A) Using **mobile phone data** to quantify connectivity between districts. Vaccination campaign averted 47% of infections in the unadjusted approach, 47% when connectivity was adjusted to Choma travel survey results, 47% when connectivity was adjusted to mixed Choma and Ndola travel survey results, and 46% when connectivity was adjusted to Ndola travel survey. B) Using the **departure-diffusion model**, fitted to mobile phone data to quantify district connectivity. The vaccination campaign resulted in 47%, 48%, 50%, and 50% reduction in cumulative infections when connectivity was estimated by unadjusted mobile phone data, mobile phone data adjusted to results of the Choma travel survey, mobile phone data adjusted to the mixed Choma and Ndola travel survey results, and mobile phone data adjusted to the Ndola travel survey, respectively.

## Figure S4. Sensitivity analysis, with the initial introduction of cases in Lusaka district only.

In this scenario, we assume that the only introductions occur in the Lusaka district. We introduce 10 cases in Lusaka in the first time step. A) Connectivity estimated using mobile phone records (unadjusted and adjusted to results from travel surveys). B) Connectivity estimated using departure-diffusion model, fit to mobile phone records (unadjusted and adjusted to results from travel surveys).


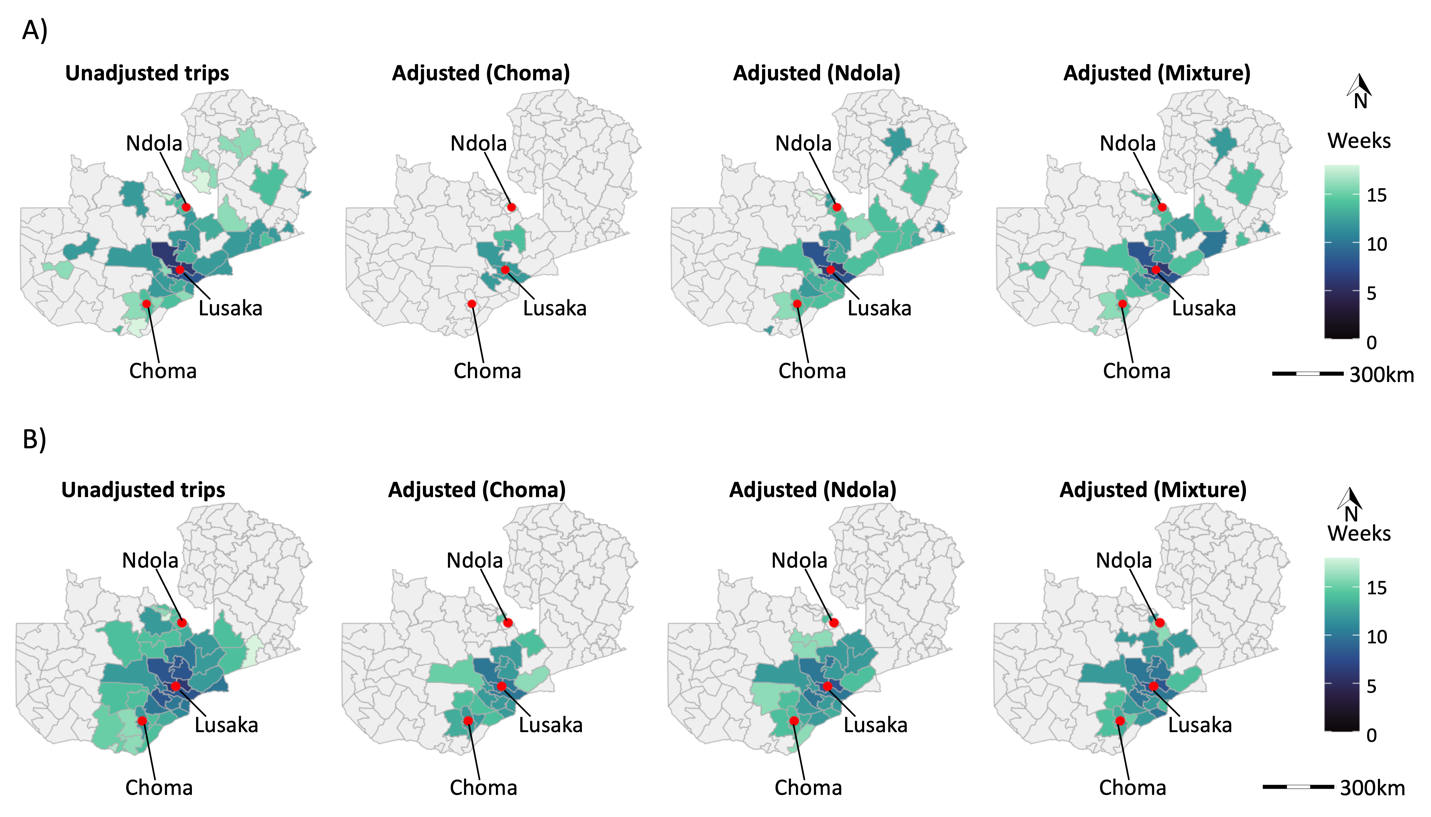


## References

1. Portnoy A, Jit M, Ferrari M, Hanson M, Brenzel L, Verguet S. Estimates of case-fatality ratios of measles in low-income and middle-income countries: a systematic review and modelling analysis. Lancet Glob Health. 2019 Apr 1;7(4):e472–81.

2. Glass K, Xia Y, Grenfell BT. Interpreting time-series analyses for continuous-time biological models--measles as a case study. J Theor Biol. 2003 Jul 7;223(1):19–25.

3. Central Statistical Office. Population and Demographic Projections, 2011-2035 - Zambia Data Portal [Internet]. 2013 [cited 2022 Oct 24]. Available from: https://zambia.opendataforafrica.org/ZMPHC2015/population-and-demographic-projections-2011-2035#

4. Ministry of Health Zambia. Post Coverage Measles-Rubella Campaign Evaluation Survey. Government of the Republic of Zambia; 2021 Nov.

5. Ministry of Health Zambia. Post MR Campaign Survey Report. Government of the Republic of Zambia; 2017 Mar.

6. Carcelen AC, Winter AK, Moss WJ, Chilumba I, Mutale I, Chongwe G, et al. Leveraging a national biorepository in Zambia to assess measles and rubella immunity gaps across age and space. Sci Rep. 2022 Jun 17;12(1):10217.
